# Capturing the complexity of healthcare for people with Down syndrome in quality indicators - a Delphi study involving healthcare professionals and patient organisations

**DOI:** 10.1186/s12913-020-05492-z

**Published:** 2020-07-27

**Authors:** Francine A. van den Driessen Mareeuw, Antonia M. W. Coppus, Diana M. J. Delnoij, Esther de Vries

**Affiliations:** 1 Tranzo, Scientific Center for Care and Wellbeing, Faculty of Social and Behavioral Sciences, Tilburg University, PO Box 90153, 5000 LE Tilburg, The Netherlands; 2 Jeroen Bosch Hospital, PO Box 90153, 5200 ME ´s-Hertogenbosch, The Netherlands; 3 Department for Primary and Community Care, Radboud University Medical Center, PO Box 9101, 6500 HB Nijmegen, The Netherlands; 4 Dichterbij, Center for the Intellectually Disabled, PO Box 9, 6590 AA Gennep, The Netherlands; 5 Erasmus School of Health Policy & Management, Erasmus University, PO Box, 3000, DR Rotterdam, The Netherlands; 6 National Health Care Institute, PO Box 320, 1110 AH Diemen, The Netherlands

**Keywords:** Down syndrome, Quality indicators, Quality of health care, Netherlands, Delphi technique

## Abstract

**Background:**

Insight into quality of healthcare for people with Down Syndrome (DS) is limited. Quality indicators (QIs) can provide this insight. This study aims to find consensus among participants regarding QIs for healthcare for people with DS.

**Methods:**

We conducted a four-round Delphi study, in which 33 healthcare professionals involved in healthcare for people with DS and two patient organisations’ representatives in the Netherlands participated. Median and 75-percentiles were used to determine consensus among the answers on 5-point Likert-scales. In each round, participants received an overview of participants’ answers from the previous round.

**Results:**

Participants agreed (consensus was achieved) that a QI-set should provide insight into available healthcare, enable healthcare improvements, and cover a large diversity of quality domains and healthcare disciplines. However, the number of QIs in the set should be limited in order to prevent registration burden. Participants were concerned that QIs would make quality information about individual healthcare professionals publicly available, which would induce judgement of healthcare professionals and harm quality, instead of improving it.

**Conclusions:**

We unravelled the complexity of capturing healthcare for people with DS in a QI-set. Patients’ rights to relevant information have to be carefully balanced against providers’ entitlement to a safe environment in which they can learn and improve. A QI-set should be tailored to different healthcare disciplines and information systems, and measurement instruments should be suitable for collecting information from people with DS. Results from this study and two preceding studies, will form the basis for the further development of a QI-set.

## Background

Down syndrome (DS) is the most prevalent genetic cause of intellectual disability (ID) [1, 2]. People with DS suffer from a large variety of health problems and therefore have complex healthcare needs, with many different healthcare providers involved [2–5].

It is widely acknowledged that healthcare for people with DS should be of high quality in order to meet their specific healthcare needs [4, 6, 7]. This is supported by the Convention on the Rights of Persons with Disabilities, advocating high-quality healthcare for people with disabilities, and acknowledging the right for obtaining the highest possible level of health [8]. However, little is known about the quality of DS-specialised healthcare [9, 10].

Quality in healthcare is multidimensional. The World Health Organization formulated six dimensions of healthcare quality: 1) effective (evidence-based and based on needs), 2) efficient (maximising resources, avoiding waste), 3) accessible (timely, geographically reasonable, in a suitable setting), 4) acceptable/patient-centred (taking into account preferences, culture of patient), 5) equitable (same level of quality for everyone) and 6) safe (minimising risk and harm) [11].

Quality indicators (QIs) - also known as quality measures [12] - are an important tool in healthcare quality, as they can improve clinical decisions, guide organisational reform, and structure the development of multidisciplinary teams [13]. Moreover, QIs can provide patients with information that enables them to choose the best suitable care [14]. However, an authors’ former study revealed that, up to now, QIs measuring quality of healthcare for people with DS, do not appear to exist [9, 10]. The study found that existing QIs concern people with ID in general (not people with DS in particular), or focus, for instance, on care in assisted living facilities (not specifically on healthcare) [9, 10].

According to Donabedian’s (2005) well-known framework for quality in healthcare, a QI-set may include different types of QIs: structure, process, and outcome QIs [13, 15]. Structure refers to the setting in which healthcare is provided (e.g. administrative structure), process to how healthcare is provided (e.g. followed procedures), and outcome to the result of healthcare provided (e.g. recovery, survival) [13]. Generally, QIs are based on quality standards, such as guidelines or protocols [16, 17]. In the Netherlands, a guideline for multidisciplinary healthcare for children with DS [18] is present and is currently being revised. Until now, such a guideline concerning adults with DS has not been present, but is currently being developed.

The present study aims to find consensus among healthcare professionals and patient organisation representatives regarding QIs for healthcare for people with DS in the Netherlands. This healthcare involves, amongst others: a paediatrician, ID physician (in the Netherlands, there is an ID-specialised training for physicians), general practitioner (GP), physiotherapist, speech therapist, psychiatrist, cardiologist, ophthalmologist, and DS-specialised multidisciplinary outpatient clinics, so-called ‘Down teams’ [3, 5, 7, 19, 20]. There are paediatric and adult ‘Down teams’ in the Netherlands. Paediatric ‘Down teams’ typically include a visit to the paediatrician, physiotherapist, ENT (ear-nose-throat)-specialist and others, all on the same day. Adult ‘Down teams’ are still scarce and have a slightly different composition, due to different needs in adulthood.

The present study is part of a larger project aiming to develop a QI-set for healthcare for people with DS. The project includes a literature review on existing QIs for healthcare for people with DS (indicating the absence of QIs that could serve as a basis for our QI-set) [10], a qualitative exploration of how people with DS, parents and support staff define quality in healthcare [21] (see Table 1), and the current study. In the final project step, findings of the three studies will be combined in order to formulate QIs. In the present study, the following research questions are addressed:

1. *According to healthcare professionals and patient organisations’ representatives, how should a QI-set measuring quality in healthcare for people with DS be defined?*
  - *Which purposes should it serve?*
  - *Which healthcare disciplines, services and quality domains should it cover?*
  - *Which type of QIs (structure, process, outcome) should it include and how many?*
2. *According to healthcare professionals and patient organisations’ representatives, what factors should be taken into account in the further development and implementation of the QI-set?*

**Table 1:** Summary of outcomes of previous study

**Outcomes from previous study**^a^	
*Method and participants:* Qualitative design including semi-structured interviews with people with DS and with parents, and focus groups with support staff members (of people with DS living in assisted living facilities)	
*Summary of findings:* - Participants mentioned a large variety of healthcare and other services people with DS used. Among others: ‘Down team’, GP, dentist, psychologist, physiotherapist, speech therapist, ear nose throat physician, ophthalmologist, family support, educational support. - According to participants, good healthcare is: o Person-centred: The person with DS and his/her values and preferences are central; The personal situation and life stage of the person with DS are taken into account and caregivers are involved; Communication between professional and person with DS (and his/her caregivers) is respectful and adapted to the abilities of the person with DS. o Effective, efficient and accessible: Timely recognition of health problems, Healthcare professionals with DS-expertise are nearby; Information about available care is present. o Multidisciplinary, well-coordinated and integrated: It includes actors outside healthcare (e.g. school, work); Information is shared (between professionals); Consultations are planned in a synchronized manner; Transition from paediatric to adult healthcare and services proceeds smoothly.	

Abbreviations: *DS* Down syndrome, *GP* General practitioner

^a^ Qualitative exploration of opinions and experiences of people with DS, parents, and support staff regarding healthcare quality [21]

## Methods

A Delphi technique was used in order to achieve consensus among experts in healthcare for people with DS about relevant items for QIs and related practical issues. Our study is an exploratory inquiry concerning personal opinions of professionals on healthcare quality. According to Dutch legislation [22], ethics approval was deemed unnecessary, since participants in our study were not subject to procedures and were not required to follow rules of behaviour. We obtained a written informed consent statement from all participants prior to the study. This allowed us to use participants’ contact details for sending them the questionnaires, or for contacting them in case of problems with receiving or filling out the questionnaires. In this statement, participants also approved the use of their answers to the Delphi-questionnaires in an anonymous manner for the aims of the study.

### Participants

We included representatives of all relevant disciplines involved in healthcare for people with DS and patient organisation representatives, all having expertise in healthcare for people with DS. This composition is similar to the composition of the working group developing guidelines for healthcare for people with DS [18]. Recruitment of participants was done by contacting professional organisations from relevant disciplines and two patient organisations (one specific DS organisation and the umbrella organisation of Dutch patient organisations). We explained the purpose of our research and the expected time investment, and asked the organisations to identify members of their organisations with expertise in healthcare for people with DS. When identified members had agreed to participate, contact details were provided to the researchers, who in turn contacted the members. As the Dutch professional organisation of GPs declined to identify eligible GPs because of other priorities, GPs were recruited via the network of the authors and participants, and/or by using publicly available contact details. Table 2 provides an overview of the participant characteristics.

**Table 2:** Participant characteristics

Characteristic	*n* = 35
Age (y) [mean (stdev) [range]]	50.5 (9.6) [30–73]
Gender [number (%)]	
Female	32 (91.4%)
Male	3 (9.0%)
Profession	
Audiologist	1 (2.9%)
Dentist (ID-specialised)	3 (8.6%)
Dermatologist	1 (2.9%)
Dietician (ID-specialised)	2 (5.7%)
General Practitioner	2 (5.7%)
ID physician	3 (8.6%)
Municipal Health Services doctor	1 (2.9%)
Nurse / coordinating nurse (ID-specialised)	3 (8.6%)
Occupational therapist	2 (5.7%)
Ophthalmologist	1 (2.9%)
Orthoptist	2 (5.7%)
Paediatrician	2 (5.7%)
(child) Physiotherapist	4 (11.4%)
Psychiatrist (child/youth/adult)	1 (2.9%)
Psychologist	1 (2.9%)
Podiatrist	2 (5.7%)
(child) Rehabilitation physician	1 (2.9%)
Representative of patient organisation	2 (5.7%)
Speech therapist	1 (2.9%)
Time working in this profession (y)	
[mean (stdev) [range]]	19.2 (10,2) [0.7–40]
Frequency of contact with people with DS [number (%)]	
(almost) daily	9 (25.7%)
Weekly	14 (40.0%)
Monthly	7 (20.0%)
Half-yearly	3 (8.6%)
Yearly	1 (2.9%)
Less than once a year	1 (2.9%)

Abbreviations: *y* year(s), *stdev* standard deviation, *ID* Intellectual Disability

### Four-round Delphi procedure

A Delphi study uses a series of questionnaire-rounds in order to establish consensus among a group of experts about a certain topic [12, 23, 24], and is suitable for the selection of QIs [25]. In such an iterative process, each next round is based on the participants’ answers in the previous round. Only items for which no consensus among participants is found, are presented in the next round. Furthermore, participants receive an overview of the overall group response of the previous round, based on which they can reconsider their initial answers [24, 25]. Our study consisted of four consecutive rounds:

- Round 1: Introduction to themes, initial inventory of level of consensus;
- Round 2: Feedback on Round 1 and revisiting themes on which no consensus existed;
- Round 3: Exploration of consensus on sub-domains;
- Round 4: Final consensus building

We used online questionnaires, which were composed using Qualtrics^XM^®. Online questionnaires allow participants to fill out the questionnaires wherever they want, allow anonymous participation of experts across various locations, and prevent one (or a few) expert(s) from dominating the consensus process [12, 23].

### Questionnaires and consensus

All questionnaires contained questions with a five point Likert-scale, multiple choice questions and open-ended questions.

Using the Likert-scale questions, participants rated items in terms of relevance for the QI-set (1 ‘very important’, 2 ‘important’, 3 ‘neutral’, 4 ‘not that important’, 5 ‘not important at all’), or indicated to what extend they agreed with propositions (1 ‘totally agree’, 2 ‘agree’, 3 ‘neutral’, 4 ‘disagree’, 5 ‘totally disagree’). In round 1 an *‘I don’t know’*-option was also included. Consensus was defined in advance, as follows: if at least 75% of the participants rated an item as 1 or 2 and the median was ≤2, consensus was achieved among the participants about including the item in the QI-set, or about agreeing with a proposition. If 75% of the participants rated an item 4 or 5 and the median was ≥4, consensus was achieved among the participants about excluding the item from the QI-set, or about disagreeing with a proposition. In all other situations, it was concluded that consensus was not achieved among participants. Although there is no standard for defining consensus in Delphi studies, using a combination of percentages and median for defining consensus is generally accepted [12, 25]. A 75% cut-off is considered adequate in Delphi studies [24]. We decided to present some items to the participants despite the fact that consensus was obtained for these items in the previous round(s), because some participants had not been able to join the first round, or because we thought the items should be presented as a complete set (e.g. all healthcare disciplines possibly involved in healthcare for people with DS). If we deemed more detailed information was needed, more specialised items/propositions, or differently formulated propositions were presented to the participants (e.g. quality domains were presented in round 1 and sub-domains in round 3).

The multiple choice questions and the open ended questions allowed participants to explain their ‘rated’ answers or add relevant QI-items.

The topics of the questionnaires were largely based on outcomes of the previous study investigating the experiences and opinions of people with DS, parents and support staff regarding quality in healthcare [21] (see Table 1) and on the multidisciplinary guideline for healthcare for children with DS [18]. Additionally, the questionnaires contained topics related to the development, implementation and use of QIs, informed by literature and expertise of the authors. Topics addressed in the questionnaires and number and type of questions are shown in Table 3. An English translation of the questionnaires can be found in Additional file 1.

**Table 3:** Topics addressed and type of questions per round

Topic addressed	Topic addressed in:			
	Round 1 Introduction to themes, initial inventory of level of consensus	Round 2 Feedback on Round 1 and revisiting themes on which no consensus existed	Round 3 Exploration of consensus on sub-domains	Round 4 Final consensus building
Participant characteristics	6 open ended questions (such as age, gender, frequency of contact with people with DS).	Idem: same questions were presented to participants who had not participated in round 1.		
Purpose of QI-set (e.g. transparency, quality improvement, auditing, insurance)	9 purposes, rate importance	12 propositions^a^	9 propositions^a^	
Quality domains to be included in QI-set (e.g. coordinated care, person-centeredness, clinical outcome)	10 items^b^ and 1 proposition for children with DS; 10 items^b^ and 1 proposition for adults with DS	7 items^b^ for children and adults with DS	28 items^b^ (sub-domains)	1 proposition^a^
Healthcare disciplines to be included in QI-set (e.g. Down team, psychological care, physiotherapy)	14 items^b^ and 1 close-ended question for children with DS; 14 items^b^ and 1 close-ended question for adults with DS	6 propositions; 30 items^b^ for children; 30 items^b^ for adults with DS	4 open-ended questions	1 proposition^a^
Number and type (structure / process / outcome) of QIs		2 close-ended questions	2 propositions; 1 close-ended question	2 propositions; 3 open-ended questions
Information sources and transparency of QIs and practical issues regarding development	1 close-ended question; 1 open-ended question	1 proposition; 1 close-ended question; 6 open-ended questions	6 propositions; 1 close-ended question; 2 open-ended question	17 propositions
Healthcare quality for people with DS and current use of QIs	3 close-ended questions; 3 open-ended questions	15 propositions		
Aim of the study	1 open-ended question			

Abbreviations: *DS* Down syndrome, *QI* Quality indicator

Empty fields indicate that the topic was not presented to the participants in the concerning round.

^a^ Participants indicated to what extent they agreed with propositions (1 ‘totally agree’, 2 ‘agree’, 3 ‘neutral’, 4 ‘disagree’, 5 ‘totally disagree’)

^b^ Participants rated items (i.e. healthcare disciplines/services or quality domains) indicating the relevance for the QI-set (1 ‘very important’, 2 ‘important’, 3 ‘neutral’, 4 ‘not that important’, 5 ‘not important at all’)

### Delphi in one day

The first questionnaire was sent out on April 25th 2018, the other three on May 30th 2018. This timeframe was chosen because participants preferred to conduct the study (predominantly) on 1 day. This short study duration would thus prevent participant drop-out related to large time intervals between the rounds. It would also limit time investment of both participants and researchers, as participants do not need re-introduction into the topic at the start of each new round, and data collection proceeds quickly. Although the time intervals between the rounds in our study were much shorter than in classic Delphi studies [24], literature does not provide any reason to assume that a shorter study duration affects the results [26]. However, in order to allow for such short time intervals, the rounds required thorough preparation, enabling participants to fill out the questionnaires swiftly, and enabling researchers to perform analyses and adapt the questionnaires accordingly. Therefore, the authors composed most questions beforehand, by anticipating the possible responses of the participants and by using preliminary insights resulting from round 1. Because of this, only a few questions needed to be newly composed between round 2, 3 and 4, and most questions only had to be moved, slightly rephrased, or removed. Additionally, used software was set ready to quickly provide the researchers with information needed to assess consensus (median and 75-percentiles) and with an overview of open-ended question answers. Furthermore, roles of the research team (i.e. obtaining medians and 75-percentiles; extracting open-ended question answers, chairing the discussions (see next paragraph “Analysis”), adapting and sending out the questionnaires) were allocated beforehand.

### Analysis

During the study, we used percentages provided by Qualtrics^XM^® and the median calculated using IBM SPSS Statistics 24, to determine whether the answers of the participants on the Likert-scale questions had resulted in consensus. From the multiple choice questions, only frequencies (percentages) were calculated. Analysis of the answers from open-ended questions included reading and discussing the answers by all authors, which resulted in identification and structuring of key issues. All authors were involved in all iterations of the study, in an e-mail conversation (first round) and in a face-to-face meeting (rounds 2–4).

Afterwards, in order to structure the data, a dataset containing data from all rounds was created using IBM SPSS Statistics 24, and median and 75-percentile of the Likert-scale questions were calculated again. The calculations were done with and without the patient organisation representatives’ answers, in order to discover whether their answers differed from the health care professionals’ answers. Differences were indicated together with the concerning findings, in order to interpret the results.

## Results

### Participants flow

**Fig. 1:**
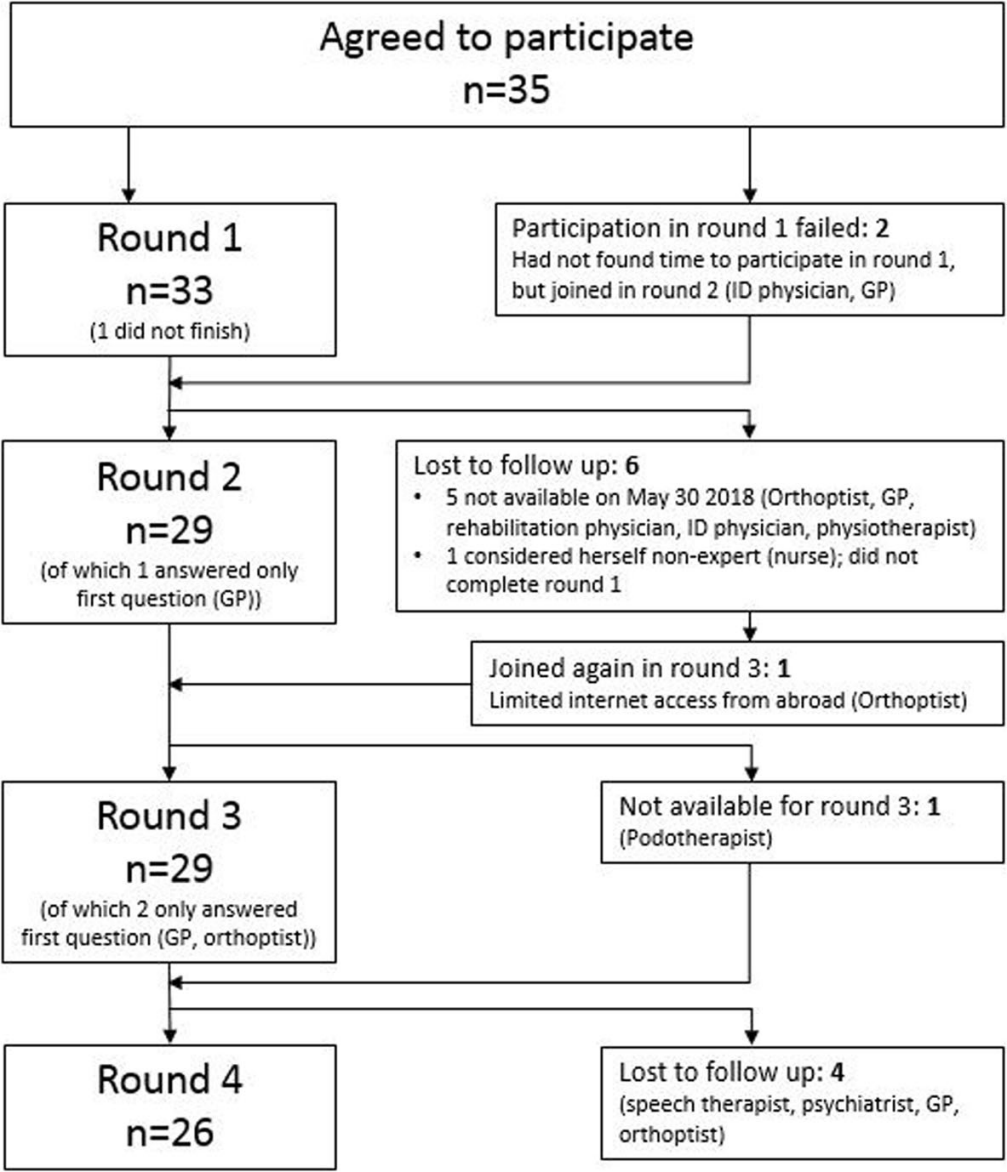
Flowchart of number of participants for each Delphi round

A total of 35 eligible participants was identified. However, one participant could not allocate time for participating in any of the rounds and answered only one question in round two and three. Ten participants could not participate in all rounds. Figure 1 shows a flowchart of the number of participants per round. On average, participants needed 55, 52, 25 and 14 min to complete questionnaires 1, 2, 3 and 4 respectively, with a maximum of 114, 85, 45, and 48 min.

### Results Delphi rounds

Distributed across the four rounds, 259 questions were presented to the participants, comprising 20 open-ended questions, 11 closed-ended questions and 228 propositions or items, of which 107 had resulted in consensus among the participants. See Table 4.

**Table 4:** Number and types of questions per round and consensus among participants on propositions and items

Round	Total number of questions	Open-ended questions	Closed-ended questions	Propositions /Items	Consensus
Round 1	72	5	6	61	37
Round 2	110	6	3	101	31
Round 3	54	6	2	46	28
Round 4	23	3	0	20	11

Below, the results of the four Delphi rounds are presented in two parts: 1) Defining purposes and identifying QI-topics; and 2) Considerations for further development and implementation of the QI-set. More details about the results can be found in Additional file 2.

#### Defining purposes and identifying QI-topics

##### Purposes

In the first three rounds, participants indicated the purpose(s) to be served by the QI-set. See Table 5, first row (‘Purpose of QIs’).

**Table 5:** Summary of findings: Defining purposes and identifying QI-topics

Theme	Consensus about (Likert-scale questions) or Majority agreed that (multiple choice / open questions)	Round(s) in which theme was addressed
Purpose of QIs	QIs should: • provide people with DS and their caregivers with information on where to find suitable healthcare (providers); • provide healthcare professionals with information on where to find suitable healthcare (providers); • be used to improve healthcare for people with DS on a national level; • be used to improve healthcare for people with DS delivered by their organisation (e.g. health centre, hospital, department), by using the QIs as input for (interdisciplinary) reflective meetings with colleagues, for short term evaluation of healthcare delivery on the patient level^a^, or for adapting protocols; • be used as input for developing guidelines; • be used for inspection and control by national/governmental or intra-organisational authorities; and • be used to reduce differences in quality of provided healthcare by different providers	1,2,3 (more detailed information in Supplementary Table 1, Additional file 1)
Quality domains	The QI-set should cover: • Coordination (both within and between organisations and disciplines) of healthcare for people with DS, including professional collaboration and agreements, and professional-caregiver collaboration; • Transition from paediatric towards adult healthcare; • Effectiveness, including expertise of healthcare professionals and timely detection of health problems; • Person-centeredness, including the social system of a person with DS^a^. • Quality of life, daily functioning, autonomy, and participation in society; • Safety; • Clinical outcomes (e.g. blood screening); and • Adherence to guidelines.	1,2,3 (more detailed information in Supplementary Table 2, Additional file 1)
Healthcare disciplines / services	• Concerning children, the QI-set should include: Down team, paediatrics, physiotherapy, speech therapy, dietetics, psychological/psychiatric care, dental hygiene, specialised dentistry, audiology (screening), and family support^b^; • Concerning adults, the QI-set should include: Down team, ID physician, dietetics, psychological/ psychiatric care, dental hygiene, palliative/geriatric care, general practitioner, audiology, and a case-manager. • QI-set should be sensitive to different healthcare needs in different life phases	1,2 (more detailed information in Supplementary Table 3, Additional file 1)
Number of QIs in set	• QIs should include all disciplines involved in healthcare for people with DS • The QI-set should contain a basic set and additional specialised modules • Each module should contain a maximum of ten QIs • Disciplines are more important to be included in the QI-set if: o more people with DS need them o they contribute more to QoL o there are more doubts about the quality provided by the discipline	2,3,4 (more detailed information in Supplementary Tables 3 and 4, Additional file 1)
Type (structure / process / outcome) of QIs in set	The QI-set should include an (almost) evenly distributed amount of structure, process and outcome QIs.	2,3 (more detailed information in Supplementary Table 4, Additional file 1)

Abbreviations: *DS* Down syndrome, *QI* quality indicator, *ID* Intellectual disability, *QoL* Quality of life

^a^ Only consensus if patient organisation representatives were left out of analysis

^b^ No consensus if patient organisation representatives were left out of analysis

Related to the purpose “provide healthcare professionals with information on where to find suitable healthcare (providers)”, participants explained that providers could use this information for making referrals. Especially for generalists (such as GPs), who cannot reasonably be expected to have much DS-specialised expertise, QIs could be helpful in identifying specialised healthcare professionals to refer to.

Additional to the purposes “improving healthcare on the national level” and “improve healthcare for people with DS delivered by their organisation (e.g. health centre, hospital, department)”, participants mentioned that QIs could be part of audits, and could be used to improve processes (logistics, management, ICT etc.). Furthermore, participants explained that QIs should enable benchmarking of one’s own functioning as compared to that of colleagues at individual, regional or national level.

About the purpose “using QIs as input for developing guidelines”, consensus was achieved in the first round. However, participants commented that QIs should not be used as input for guidelines, but rather the other way around (guidelines should define indicators). We therefore decided to present this purpose to the participants in round two again, which did not result in consensus.

Although there was consensus concerning “QIs should be used to reduce differences in quality of provided healthcare by different providers”, some participants argued that differences should exist between providers, because if differences would not exist, this may imply that differences between centres of expertise and other healthcare providers - very much needed for healthcare for people with DS – could not exist.

##### Quality domains

In the first three rounds, participants indicated per quality (sub-)domain how important they considered it to be covered by the QI-set. Table 5, second row (‘Quality domains’) shows the quality domains that, according to consensus among the participants, should be covered by the QI-set.

Although consensus existed regarding including person-centeredness in the QI-set, this was not reflected in participants’ answers regarding sub-domains of person-centeredness, presented to the participants in following rounds. On the one hand, participants explained that QIs should measure whether healthcare is adapted to the needs of the person with DS, which may also increase effectiveness. On the other hand, no consensus existed about: adapting care to the preferences and desires of the person with DS, self-management, considering experienced burden for parents and other caregivers, and organising multidisciplinary appointments on 1 day.

Furthermore, participants argued that concepts such as quality of life and daily functioning should not appear in the QI-set, because they are too complex to be measured by QIs, too little related to quality of delivered care, or more suitable for inclusion in scientific research, than for being part of a QI-set. Others argued that such concepts should appear in the QI-set, because this would result in increased awareness among healthcare professionals about these important concepts.

##### Healthcare disciplines/services

In round one and two, participants indicated how important they considered each healthcare discipline or service to appear in the QI-set (see Table 5, third row (‘Healthcare disciplines / services’)). Participants unanimously indicated that the set should contain one or more QIs on Down teams for children. It was even argued that a QI for Down teams could function as an indicator for the quality of all other healthcare for a child with DS, because Down teams are expected to have an overview over the total package of care. However, it was also noted that not all children with DS visit Down teams, implying that a ‘Down team QI’ would not be able to indicate quality of healthcare for all children with DS. A QI measuring quality of care provided by a paediatrician would therefore be more important. Similarly, a QI measuring healthcare quality of adult Down teams, would not be representative for all healthcare for adults with DS, since the number of adult Down teams is (too) small, as is the number of ID physicians. Participants explained that GPs sometimes provide the healthcare that is not provided by ID physicians / adult Down teams. Therefore, including a QI on healthcare provided by GPs could be important for adults with DS. However, a reason mentioned for *not* including GP-care in the QI-set is that GPs were not expected to have DS-expertise, because they have only a small number of patients with DS.

Furthermore, participants did not agree about coverage of visual functioning and dental care. Monitoring visual functioning was mentioned as a candidate indicator, because visual functioning is apt to change over time. However, no consensus was achieved on including visual screening in the set. Participants’ comments about dentistry indicated that some sort of dentistry should be in the QI-set. However, it remains unclear which form of dentistry should be in the QI-set, as some people with DS need a specialised dentist, while for others a general dentist suffices. A mentioned reason for including a QI measuring specialised dental care, was based on the idea that a specialised dentist should always be involved, in order to monitor, recognise and treat DS-specific dental problems.

There was a lot of discussion about including non-medical disciplines/services in the QI-set. For example, consensus about including ‘family support’ was only achieved when the patient organisations’ representatives were included in the analysis, and there was no consensus about including support staff of assisted living facilities in the QI-set. Moreover, the proposition “QIs should also cover non-medical disciplines” did not result in consensus. Some participants argued that including them was especially important because it is too much of a blind spot among healthcare professionals, whereas others explained that non-medical disciplines/services do not belong to a QI-set for quality of healthcare.

Although participants considered adherence to medical guidelines to be an important QI, they also noted that deviation from guidelines may be necessary in order to provide care that answers to the needs of people with DS. Hence, non-adherence to guidelines does not necessarily indicate low quality.

##### Number and type of QIs

Table 5, fourth row (‘Number of QIs in set’) shows that participants preferred to include all disciplines/services involved in healthcare for people with DS in the QI-set. However, participants also noted that this would result in a QI-set with too many QIs, leading to a too high administrative burden for the users of the QI-set. In round two, participants thought that the total number of QIs in the set should be, or should not exceed, ten. In round three, participants agreed (consensus) that the QI-set should consist of modules: a basic module containing QIs relevant for all people with DS, and additional modules for specific patient groups or healthcare services. In round four, participants thought that each module should contain about ten QIs.

In round two and three, participants indicated that they thought the QI-set should contain structure, process, and outcome QIs (see Table 5, fifth row (‘Type of QIs in set’)). They also argued that the number of outcome indicators should be the highest, followed by process and structure indicators respectively.

#### Considerations for further development and implementation of the QI-set

##### Current and future use of indicators

In round one, the majority of the participants indicated that they expected their colleagues (from the same profession) to be willing to register (extra) data for the QI-set. See Table 6, first row (‘Willingness to register’). Participants explained that whether or not healthcare professionals would register data for this QI-set, would be dependent on available time, awareness about the QIs, considered utility of QIs, and frequency of contact with people with DS.

**Table 6:** Summary of findings: current and future use of indicators

Theme	Answers to multiple choice / open questions (first 4 rows) and one Likert-scale question (last row)	Number (%) of participants	Round(s) in which theme was addressed
Willingness to register	- My colleagues (from the same profession) will not be willing to register (extra) data for the QI-set	5^a^ (16%)	1 (*n* = 32)
	- My colleagues will only be willing to register (extra) data for the QI-set if this would only mean ‘clicking a few extra boxes’	14^b^ (44%)	
	- My colleagues will be willing to register (extra) data.	13^c^ (41%)	
Current collection of data by own organisation	- Information on adherence to guidelines	10 (31%)	1 (*n =* 32)
	- Transition from paediatric to adult healthcare	3 (9%)	
	- Clinical outcomes	10 (31%)	
	- Quality of life / daily functioning / participation	9 (28%)	
	- Coordination within the organisation	5 (16%)	
	- Coordination between organisations/ disciplines	1 (0%)	
	- Whether organisation is findable for potential patients	4 (10%)	
	- Accessibility	6 (19%)	
	- Expertise of healthcare professionals	7 (22%)	
	- Person-centeredness	9 (19%)	
	- Equity	4 (10%)	
	- No quality information collected	13 (41%)	
	- N/A	5 (16%)	
Current use of QIs	- Indicators regarding general internal improvement of healthcare (non DS-specific) or audits,	11 (34%)	1 (*n =* 32)
	- Indicators regarding client satisfaction,	6 (19%)	
	- Indicators regarding discipline/condition-specific (non DS-specific) issues	4^g^ (13%)	
	- No indicators	11 (34%)	
	- N/A	2^h^ (6%)	
Current use of guidelines	- The multidisciplinary medical guideline for children with DS	13 (38%)	1 (*n =* 32)
	- A general guideline for adults with DS, developed by the organisation I work for	2 (6%)	
	- Discipline-specific guideline(s) for the general population	7^d^ (22%)	
	- Discipline-specific guideline(s) for people with ID	4^e^ (13%)	
	- Discipline-specific guideline(s) for people with DS	7^f^ (22%)	
	- No guidelines	4 (13%)	
Transparency	- QIs should provide quality information on departmental or organisational level (not on individual professionals’ level) - Providers should be obliged to publish this quality information on their websites, if they want to be seen as ‘DS-specialised’. - QIs should stimulate healthcare improvement, not judge healthcare professionals - Privacy of professionals should be protected just as much as privacy of patients.	Percentages are not applicable: consensus was achieved	3 (*n =* 29), 4 (*n* = 26) (more detailed information in Supplementary Table 5, Additional file 1)

Abbreviations: *DS* Down syndrome, *QI* quality indicator, *ID* Intellectual disability

^a^ child physiotherapist, dermatologist, GP, ID physician, psychiatrist

^b^ audiologist, 2 podiatrists, ID physician, ID-specialised dentist, municipal health services doctor, 2 occupational therapists, ophthalmologist, 2 orthoptists, paediatrician, rehabilitation specialist, speech therapist

^c^ 2 dieticians, 2 ID-specialised dentists, 2 ID-specialised nurses, paediatrician, 3 (child) physiotherapists, psychologist, and the two patient organisation representatives

^d^ GP, occupational therapy, dermatology

^e^ dentistry, dietetics, dementia

^f^ physiotherapy for children, speech therapy for children, municipal health service

^g^ dentistry, dermatology, cataract, thyroid

^h^ One of the two patient organisation representatives and one retired participant

In round one, we also asked participants what kind of quality information they or their organisation currently collected. See Table 6, second row (‘Current collection of data by own organisation’). Most participants (41%) indicated that their organisation did not collect any quality information. If information was being collected, it primarily concerned information about adherence to guidelines, clinical outcomes, and findability of the organisation. Furthermore, most participants indicated that they did not use indicators in their work, and if they did use them, it concerned QIs regarding general (not DS-specific) internal improvement of healthcare or audits (see Table 6 third row (‘Current use of QIs’)). We also asked participants about the guidelines they currently used in their work (see Table 6, fourth row (‘Current use of guidelines’)). The Dutch multidisciplinary medical guideline for children with DS [18] was the most often mentioned guideline.

Participants were not always in favour of participating in a QI-set that would make quality information publicly available, especially if a QI-set would reveal quality information on the level of individual healthcare professionals. In round one, participants explained that such information would possibly result in long waiting lists for ‘good’ providers or professionals, which may in turn negatively affect quality. Moreover, once a healthcare provider or professional is labelled as ‘not good’, this would possibly affect the choice of patients for this provider or professional for a long period of time. Because of these considerations, clarifying propositions were presented to the participants in rounds three and four (see Table 6, last row (‘Transparency’)). This confirmed the reluctance of participants to publish quality information (provided by the QIs) about individual professionals. It also showed that participants preferred access to this individual information to be limited to healthcare providers, in order to prevent judgement of healthcare professionals by patients or other parties. It should be used for internal improvements instead. Accordingly, participants explained to be reluctant to introduce a quality mark for healthcare providers. However, other participants argued that a QI-set would enable healthcare providers/organisations to profile themselves as ‘good’ healthcare providers, by ‘signing up’ for participating in the QIs, on a voluntary basis. Participation in the QI-set would be an indication of DS-expertise, which would also provide insight into available healthcare for people with DS to caregivers and healthcare professionals.

##### Data source and development of QIs

Electronic medical records (EMRs) and patient/parent questionnaires were considered the most important information sources for the QI-set. At the same time, participants underlined that both healthcare professionals and people with DS and their caregivers should not be overcharged with registration burden. See Table 7, first row (‘Data source’). Participants suggested to transform (a) patient/parent questionnaire(s) into an easy-to-understand app in order to make it suitable for people with DS. Ideally, such an app should be linked to the information system (EMR) in order to store all information together. However, participants identified the large number of existing information systems, often not mutually communicating, as a potential barrier for implementation of a QI-set.

**Table 7:** Summary of findings: data source and development of QIs

Theme	Answers to multiple choice / open questions (rows 1 & 3) and one Likert-scale question (row 2)	Number (%) of participants	Round(s) in which theme was addressed
Data source	- Data for the QIs should be extracted from the electronic medical records of patients	26 (81%)	1 (*n =* 32)
	- Data for the QIs should be obtained via questionnaires for patients/parents.	25 (78%)	
	- Burden for people with DS and their caregivers should be as low as possible when measuring quality; - People with DS/caregivers as well as healthcare professionals should deliver information for the QIs; - Parents/other caregivers should themselves be responsible for documenting and keeping track of needed healthcare for the person with DS; - When people with DS are not able to provide quality information themselves, their legal representative should decide who is eligible to provide this information. - A dialogue between healthcare professional and person with DS can be used as instrument for measuring customer satisfaction^a^	Percentages are not applicable: consensus was achieved	4 (*n =* 26) (more detailed information in Supplementary Table 5, Additional file 1)
Development of QIs	- With involvement of people with DS	23 (83%)	2 (*n* = 28)
	- With involvement of parents/caregivers	26 (93%)	
	- With involvement of healthcare professionals	27 (97%)	
	- With involvement of health insurers	6 (21%)	
	- I am willing to participate in development	9 (31%)	
	- Whether I am willing to participate depends on the time and effort needed for participation	17 (59%)	
	- I am not willing to participate	3 (10%)	

Abbreviations: *DS* Down syndrome, *QI* quality indicator, *ID* Intellectual disability

^a^ There was only consensus among the participants about this proposition if the patient representatives were left out of the analysis

According to the participants, development of the QIs should be done by researchers (the authors) together with all stakeholders. See Table 7, second row (‘Development of QIs’). Participants mentioned representatives of the same diversity of disciplines as mentioned under ‘healthcare disciplines/services’ to be involved in the development of the QIs. It was also noted that it would be difficult to weigh the different opinions of those involved. The majority of the participants (59%) indicated that whether or not they themselves were willing to participate in development of the QIs depended on the time and effort needed.

## Discussion

In this study we aimed to prefigure quality indicators for healthcare for people with Down syndrome. We used a Delphi technique involving healthcare professionals and patient organisations’ representatives. The findings of this study, together with findings from two previous studies of the authors (a literature review on existing QIs and a qualitative study involving people with DS and their caregivers [10, 21]), will be used to inform the further development and implementation of the QI-set.

According to the participants in the current study, QIs should be suitable to inform healthcare quality improvement, and should be able to provide an overview of available healthcare to people with DS and their caregivers, and to healthcare professionals. Participants stressed that QIs should not be used to judge healthcare professionals. Furthermore, they opted for an evenly distributed mix of structure, process, and outcome QIs, covering the following quality domains: coordination and continuity of healthcare, effectiveness, safety, person-centeredness, and outcomes concerning health and quality of life. Additionally, participants argued that the QIs should cover all healthcare disciplines involved in healthcare for people with DS. However, they urged to keep the number of QIs low, in order to prevent (administrative) burden for healthcare professionals and people with DS and/or caregivers. Furthermore, development of QIs should be done with involvement of all relevant stakeholders.

### Quality improvement and well-informed choices

According to the participants in our study, two key purposes of a QI-set for healthcare for people with DS are 1) to improve quality in healthcare and 2) to increase insight into available healthcare, enabling people with DS (and their caregivers) to make well-informed healthcare choices, and supporting healthcare professionals to make well-informed referrals. However, participants in the current study argue that the two purposes may conflict with each other. They explained that if quality information was publicly available, especially when it concerned information on the level of individual providers, a “shaming-and-blaming” situation would emerge. They were concerned that this would hamper quality of care, instead of improve it. A study addressing Parkinson’s disease, showed a similar reticent attitude amongst healthcare professionals towards sharing quality information with patients [27]. On the other hand, current movements in practice and literature have shown the need for encouraging patients to make well-informed healthcare choices, although the influence of QIs on healthcare choices made by patients has been shown to be limited [27–29]. Hence, patients’ rights to relevant information, fostering the choice for the best suitable healthcare, have to be carefully balanced against providers’ entitlement to a safe environment in which they can learn and improve.

### Capturing complexity

There was much discussion about defining the coverage of the QI-set. Some participants preferred to include only medical QIs, whereas others were convinced that a QI-set should cover disciplines/services outside healthcare, such as support staff of assisted living facilities, in order to reflect the complexity of healthcare for people with DS [5, 30]. However, based on our results (achieved consensus) we conclude that participants prefer to limit the coverage of the QI-set to the medical domain (including psychological care). This medical focus may be a reflection of the specialised focus of healthcare professionals and their training, or of the fragmented care system in the Netherlands [31, 32]. Another explanation for this medical focus may be found in social psychology [33, 34]: healthcare professionals may consider quality improvement or transparency within the medical domain within their control, while they consider other domains beyond their sphere of influence and therefore less important for a QI-set. The medical focus may however also be a result of the participants’ reluctance to face a high registration burden, which participants repeatedly expressed during the study. This confirms the general understanding that QI-sets should be concise to foster their actual use [35, 36].

However, even if the coverage of the QI-set will be limited to the medical domain, it will, due to the multi-morbidity related to DS [5, 30], include a lot of different disciplines, and many quality domains. Hence, developing a concise QI-set will be challenging, even more so as not all quality domains may be applicable to all disciplines and contexts, and the QI-set will have to be compatible with a large variety of data registration systems used by the different healthcare providers involved. In order to limit registration burden, registration of data for a QI-set should be possible together with other currently registered data in the electronic medical record (EMR). This would also prevent registration of the same data in separate registries [37], and facilitate data collection (i.e. extraction from information systems) for the QI-set. Literature shows that automated extraction of indicators from EMRs is possible, however, the structure of information systems and the accuracy of registration by professionals is not always sufficient for enabling automated extraction [38, 39]. Nevertheless, most participants in our study thought that their colleagues (of the same profession) would be willing to register extra QI-data, especially if registration efforts would be kept as small as possible.

### Patient reported information

Participants also suggested to use patient reported information (for example from questionnaires) as input for the QI-set, which should ideally be stored within the EMR, together with the data registered by healthcare professionals. Such patient information is often obtained using Patient Reported Outcome Measures (PROMs) and/or Patient Reported Experience Measures (PREMs) [40, 41]. PROMs focus on measuring outcomes of treatments related to patient functioning, while PREMs address patient experiences regarding healthcare processes [36, 39]. PREMs/PROMs are considered robust quality measures [41]. However, due to their cognitive abilities [4], people with DS may not always be able to provide patient reported information, in which case proxies (such as parents) will have to provide this information [42, 43]. Nevertheless, patient involvement in healthcare is considered increasingly important in delivering high quality healthcare in general [44], and concerning people with ID [45]. It may therefore be worthwhile to explore other ways to obtain information from people with DS that could be used for quality improvements. Examples are using narratives for evaluation [46] or apps especially designed for people with DS/ID [47].

### Strengths and limitations

The selection of participants reflected the large variety of healthcare providers involved in healthcare for people with DS and included two patient organisations’ representatives. Although this presumably led to heterogeneity in answers, which may complicate the formulation of QIs, it can be considered a strength of the study. Participant heterogeneity enriches the results of a Delphi study, which enhances the credibility and acceptance of resulting QIs [12].

Another strength of the study is that consensus was defined in advance [12, 24, 25] (median ≤ 2 in combination with a 75% cut-off).

The fact that the members of the research team (i.e. the authors) have been collaborating before, may have led to some advantageous knowledge of each other’s ideas, which may have affected the research team’s discussions, and in turn, the content validity of the Delphi-questionnaires. However, we expect this effect to be small because of the heterogeneity of the research team (see “Authors’ information”) and the limited contact frequency of the team members before the study. Moreover, the fact that consensus was defined in advance, improves reliability of the questionnaire results.

There was variation among the participants regarding the time they had been working in their current position, but they represented ample DS-related experience: 91.4% of the participants had been working in their current position for more than 7 years; 85.7% had at least monthly contact with clients with DS.

Unfortunately, GPs, playing a key role in healthcare for people (especially adults) with DS [48], were underrepresented. Despite extensive attempts, we were only able to include one GP, who could only participate in round one.

The time intervals between the rounds in our study were much smaller than in classic Delphi studies, which have a total study duration of three to twelve months [24]. The short time-intervals were chosen after consulting the participants about their preferences for taking part in the study, in order to limit participant drop-out. Nevertheless, we could not prevent a drop-out of about 25%. However, a response rate of about 75% is considered quite high in Delphi-studies [24]. This relatively high rate was probably achieved by the personal touch we applied in communication with our participants, which is mentioned to be crucial in limiting drop-out [24]. A possible disadvantage of the short time intervals may be that it entails limited time for analysis and preparation of questions for next rounds. We mitigated this possible effect on data collection and results by preparing a large part of the questions for successive rounds in advance. Another possible disadvantage of short time intervals is related to the fact that participants have less time to reflect on, and adapt, their answers. However, we considered the questionnaires suitable to be completed within short time intervals, as the complexity of the questions presented to the participants was quite low. This is supported by the fact that the participants in our study completed the questionnaires within reasonable time. Moreover, the most complex questions, which may require much reflection time, were placed in the first questionnaire, which participants had to complete within several weeks (instead of within several minutes for the other questionnaires).

## Conclusions

Our study showed the complexity of capturing healthcare for people with DS in a QI-set that is relevant for both healthcare providers and people with DS plus their caregivers. We have taken a solid step in unravelling this complexity and its possible impact on developing QIs, thereby making substantial progress in the development of QIs for healthcare for people with DS. Future research can (and will) build further on this foundation.

Since our study involves a large variety of healthcare professionals, with heterogenic view points, our findings may not only be relevant to healthcare for people with DS, but probably to any healthcare discipline. It is even argued that, because of the complexity of healthcare for people with DS, the DS population could be used to assess the quality of the healthcare system in general [2].

Several important lessons from this study should be taken into account in the further development of a QI-set for healthcare for people with DS. First, our findings indicate that a QI-set for healthcare for people with DS has two main purposes: it should be suitable for 1) identifying possibilities for improvement of healthcare for people with DS; and 2) for supporting patients and providers in choosing appropriate healthcare (providers). However, the two purposes need to be carefully balanced, as extensive information transparency fostering patients’ healthcare choices, may conflict with ensuring safe and supportive working environments for healthcare professionals, and with fair comparison of providers. Second, capturing healthcare for people with DS in a QI-set requires the set to be suitable for use by all different disciplines involved, and to be compatible with different information systems. At the same time, the set has to be as concise and compact as possible, in order to limit administrative burden. Third, measurement instruments providing information for a QI-set should be suitable for collecting information from people with DS and their caregivers.

## Supplementary information

**Additional file 1.** (Questionnaires). English translation of the questionnaires of round 1, 2, 3 and 4. This document contains an English translation of the online questionnaires (in Dutch) that were used in the four Delphi-rounds for data collection.

**Additional file 2: **(Supplementary Tables 1–5). Supplementary Tables 1–5: **Supplementary Table 1.** Extent to which consensus was achieved among participants regarding: Purposes of QIs; **Supplementary Table 2.** Extent to which consensus was achieved among participants regarding: Quality domains; **Supplementary Table 3.** Extent to which consensus was achieved among participants regarding: Healthcare services/disciplines; **Supplementary Table 4.** Preferred number and type of QIs and extent to which consensus was achieved among participants regarding related propositions; **Supplementary Table 5.** Extent to which consensus was achieved among participants regarding: Information sources and transparency of QIs and practical issues regarding development. Tables indicating the extent to which consensus was achieved among participants regarding different aspects of a QI-set for healthcare for people with DS. Results of the study are largely based on these data.

## Data Availability

The datasets used and analysed during the current study are available from the corresponding author on reasonable request. However, most data generated or analysed during this study are included in this published article and its supplementary information files.

## Abbreviations

DS: Down syndrome
EMR: Electronic medical record
ENT: Ear Nose Throat
GP: General practitioner
ID: Intellectual disability
PREM: Patient reported experience measure
PROM: Patient reported outcome measure
QI: Quality indicator
QoL: Quality of life
Stdev: Standard deviation
