# Supplementary material for: Capturing the complexity of healthcare for people with Down syndrome in quality indicators - a Delphi study involving healthcare professionals and patient organisations
Source: BMC Health Serv Res. 2020 Jul 27;20:694. doi: 10.1186/s12913-020-05492-z (PMC7385945; doi:10.1186/s12913-020-05492-z)
Supplement: Supplementary file 1 — Additional file 1. (Questionnaires). English translation of the questionnaires of round 1, 2, 3 and 4. This document contains an English translation of the online questionnaires (in Dutch) that were used in the four Delphi-rounds for data collection. [file 12913_2020_5492_MOESM1_ESM.docx]

**Additional file 1**

***English translation of the questionnaires of round 1, 2, 3 and 4.***

*(Original questionnaires in Dutch)*

Round 1

Dear participant,

Thank you very much for your willingness to take part in this study. This study concerns quality indicators for measuring quality of healthcare for people with Down syndrome. The aim of this study is to identify potential quality indicators and to reveal how these indicators may be used in practice. The study involves four rounds: the current one and three on May 30. The first round entails the current questionnaire, which you are about to start in a few clicks. Please complete this first questionnaire before May 14.

You gave informed consent for participation in this study. Please note that participation is on a voluntary basis. You are free to withdraw from the study at any moment, without an explicit reason.

Please do not hesitate to contact us if you experience any problems [phone number and e-mail address of first author].

Good luck!

Kind regards,

[names of the authors]

Please click “next” to start the questionnaire.

1. What is your age? (scroll down menu 20-90)

2. Wat is your gender?

- Male
- Female

3. What is your current professional position?

*If you are currently not employed, please mention this in your answer and indicate the position you have had for the longest period.*

…

4. (approximately) how long did you work / have you been working in this position?

*If you are currently not employed, please indicate how long you worked in the position indicated in the former question.*

…

5a. Professionally, how often are you in contact with people with Down syndrome (children and/or adults)?

*If you are currently not employed, please indicate how often you were professionally in contact with people with Down syndrome in the position mentioned in the former question.*

- (almost) daily
- Weekly
- Monthly
- Biannually
- Annually
- Less than once a year

5b. Explanation related to your profession and/or your contact with people with Down syndrome (questions 3-5)

(optional)

…

Indicators

Based on literature, existing guidelines, previous input of healthcare professionals and interviews with people with Down syndrome, their parents, and support staff, we identified relevant elements of quality of healthcare for people with Down syndrome. The number of elements appeared to be large.

*"An indicator is a ‘measurable element of practice performance (…) that can be used to assess the quality, and hence change in quality, of care provided” (Lawrence et al., 1997). An indicator is a signaling agent: it is not a direct measure of quality, but indicates a certain aspect of healthcare provision, which may be reason for further investigation. (Handleiding indicatorenontwikkeling, Kennisinstituut Medisch Specialisten, 2013)*

Preferably, a small number of indicators provides as much information as possible. In other words, we strive to obtain a good impression of quality of healthcare for people with Down syndrome using only a few indicators. An important reason for this is to limit the administrative burden. Therefore, this study aims to select indicators that best reflect healthcare quality, and at the same time, lead to the least administrative burden. However, this study starts extensively, with a broad variety of topics to be potentially measured by the indicators. With the following questionnaires, we aim to reveal the topics that are, according to you, relevant for a set of indicators for healthcare for people with Down syndrome. In the following questions, a large variety of topics is presented to you. For each topic, you are asked to indicate how important you think it is (the extent to which you think the topic should be reflected in the set of indicators). You are asked to do this for healthcare for both children (0-17 years of age) and adults (18 years of age and older). You will be able to explain your answers if desired.

The following questions concern healthcare for CHILDREN (0-17 years of age) with Down syndrome.

6. Healthcare for children (0-17 years of age) with Down syndrome:

6a. Which elements of healthcare should be reflected in the set of indicators?

|  | Very important | Important | Neutral | Not that important | Not important at all | I don’t know | Explain your answer (optional) |
| --- | --- | --- | --- | --- | --- | --- | --- |
| Down team  (a coordinated team of collaborating multidisciplinary healthcare providers for children with Down syndrome (0-17y)) | O | O | O | O | O | O | … |
| Pediatrician | O | O | O | O | O | O | … |
| Physiotherapy | O | O | O | O | O | O | … |
| Speech therapist | O | O | O | O | O | O | … |
| Dietician | O | O | O | O | O | O | … |
| Occupational therapy | O | O | O | O | O | O | … |
| Podiatrist | O | O | O | O | O | O | … |
| Dermatology | O | O | O | O | O | O | … |
| Mental healthcare | O | O | O | O | O | O | … |
| Youth healthcare (municipal health service) | O | O | O | O | O | O | … |
| Dental care: Dental hygienist | O | O | O | O | O | O | … |
| Dental care: regular dentist (primary care) | O | O | O | O | O | O | … |
| Dental care: pediatric / specialised dentist | O | O | O | O | O | O | … |
| General practitioner | O | O | O | O | O | O | … |

6b. Which additional elements of healthcare should be reflected in the set of indicators? (in other words, do you miss elements of healthcare / disciplines in the above list and how important do you consider them to be?

PLEASE NOTE: this question (still) concerns children with Down syndrome. (questions concerning adults will follow)

(If you do not want to add elements of healthcare / disciplines, please click “next”).

| Add healthcare element(s) below | Very important | Important | Neutral | Not that important | Not important at all | Explain your answer (optional) |
| --- | --- | --- | --- | --- | --- | --- |
| … | O | O | O | O | O | … |
| … | O | O | O | O | O | … |
| … | O | O | O | O | O | … |
| … | O | O | O | O | O | … |
| … | O | O | O | O | O | … |

7. Healthcare for children (0-17 years of age) with Down syndrome:

7a. Do you think the set of indicators should be able to measure adherence to the NVK-guideline?

(We mean the guideline of the Dutch Pediatric society (NVK) of 2011: “Een update van de mulitdisciplinaire richtlijn voor de medische begeleiding van kinderen met Downsyndroom” [An update of the multidisciplinary guideline for the medical support of children with Down syndrome]. This guideline is currently being revised. We assume that your answers also apply to the revised version of the guideline. Please find the guideline and its summary here. [weblink to guideline]

|  | Very important | Important | Neutral | Not that important | Not important at all | I don’t know |
| --- | --- | --- | --- | --- | --- | --- |
| The indicator set should be able to measure adherence to the NVK-guideline | O | O | O | O | O | O |

[IF answer to 7a. was “I don’t know”, “not important at all”, or “not that important”:]

7b. Please explain your answer

…

[IF answer to 7a. was “very important”, “important”, or “neutral”:]

7c. Which elements of the NVK-guideline should be reflected in the indicator set? (more than one answer possible)

- Visit to a pediatrician (frequency with which a child with down syndrome (and his/her parents) visits a pediatrician)
- Visit to a Down team (frequency with which a child with down syndrome (and his/her parents) visits a Down team)
- Visit to an ENT-physician (frequency with which a child with down syndrome (and his/her parents) visits an ENT-physician)
- Visit to an ophthalmologist (frequency with which a child with down syndrome (and his/her parents) visits an ophthalmologist)
- Visit to an orthoptist (frequency with which a child with down syndrome (and his/her parents) visits an orthoptist)
- Visit to a dentist (frequency with which a child with down syndrome (and his/her parents) visits a dentist)
- Visit to an orthodontist (frequency with which a child with down syndrome (and his/her parents) visits an orthodontist)
- Visit to a physiotherapist (frequency with which a child with down syndrome (and his/her parents) visits a physiotherapist)
- Visit to a speech therapist (frequency with which a child with down syndrome (and his/her parents) visits a speech therapist)
- Visit to youth care (frequency with which a child with down syndrome (and his/her parents) visits youth care)
- Whether a cardiac ultrasound is made
- Thyroid screening
- Celiac disease screening
- Other elements / topics, namely: ……
- I don’t know

[IF answer to 7a. was “very important”, “important”, or “neutral”:]

7d. Please explain your answer(s) to question 7c. (optional)

…

8. Healthcare for children (0-17 years of age) with Down syndrome:

8a. Do you think that the set of indicators should include indicators reflecting the use of additional guidelines, protocols and / or quality standards?

|  | Very important | Important | Neutral | Not that important | Not important at all | I don’t know |
| --- | --- | --- | --- | --- | --- | --- |
| The indicator set should be able to measure adherence to additional guidelines / protocols / quality standards | O | O | O | O | O | O |

[IF answer to 8a. was “very important”, “important”, or “neutral”:]

8b. Which additional guidelines / protocols / quality standards?

(For example: guidelines in your field of expertise, or from the Dutch quality framework for care for people with intellectual disabilities, or other)

…

8c. Please explain (optional)

…

9. Healthcare for children (0-17 years of age) with Down syndrome:

9a. Which quality domains or topics should be reflected in the set of indicators?

|  | Very important | Important | Neutral | Not that important | Not important at all | Explain your answer (optional) |
| --- | --- | --- | --- | --- | --- | --- |
| Clinical outcomes  (for example: improved serology, cardiac function, BMI) | O | O | O | O | O | … |
| Outcomes relevant for the patient (e.g. quality of life, daily functioning, participation) | O | O | O | O | O | … |
| Coordination *within* an organization or department (for example: presence of a coordination, multidisciplinary consultation) | O | O | O | O | O | … |
| Coordination *between* healthcare professionals of different organizations and sectors (for example: between healthcare professionals in primary care, secondary care, or social care) | O | O | O | O | O | … |
| Transition 18- to 18+  (transition / transfer from pediatric to adult healthcare) | O | O | O | O | O | … |
| Findability (information on available healthcare providers and their differences) | O | O | O | O | O | … |
| Accessibility (for example: ease of making appointments, waiting time, geographical location) | O | O | O | O | O | … |
| Expertise (for example knowledge present among healthcare professionals) | O | O | O | O | O | … |
| Person-centeredness (for example: patient-professional relation, communication, taking into account life phase and preferences of patient and parents) | O | O | O | O | O | … |
| Equality | O | O | O | O | O | … |

9b. Which additional quality domains or topics should be reflected in the set of indicators? (in other words, do you miss quality domains or topics in the above list and how important do you consider them to be?

PLEASE NOTE: this question (still) concerns children with Down syndrome. (Questions concerning adults will follow)

(If you do not want to add quality domains or topics, please click “next”).

| Add quality domains or topics below | Very important | Important | Neutral | Not that important | Not important at all | Explain your answer (optional) |
| --- | --- | --- | --- | --- | --- | --- |
| … | O | O | O | O | O | … |
| … | O | O | O | O | O | … |
| … | O | O | O | O | O | … |
| … | O | O | O | O | O | … |
| … | O | O | O | O | O | … |

The following questions concern healthcare for adults (18 years and older) with Down syndrome.

10. Healthcare for adults (18 years and older) with Down syndrome:

10a. Which elements of healthcare or disciplines should be reflected in the set of indicators?

|  | Very important | Important | Neutral | Not that important | Not important at all | I don’t know | Explain your answer (optional) |
| --- | --- | --- | --- | --- | --- | --- | --- |
| Down team  (a coordinated team of collaborating multidisciplinary healthcare professionals for adults with Down syndrome (≥18y)) | O | O | O | O | O | O | … |
| ID (intellectual disability) physician | O | O | O | O | O | O | … |
| Psychologist | O | O | O | O | O | O | … |
| Dietician | O | O | O | O | O | O | … |
| Physiotherapy | O | O | O | O | O | O | … |
| Speech therapist | O | O | O | O | O | O | … |
| Occupational therapy | O | O | O | O | O | O | … |
| Podiatrist | O | O | O | O | O | O | … |
| Dermatology | O | O | O | O | O | O | … |
| Mental healthcare | O | O | O | O | O | O | … |
| Youth healthcare (municipal health service) | O | O | O | O | O | O | … |
| Dental care: Dental hygienist | O | O | O | O | O | O | … |
| Dental care: regular dentist (primary care) | O | O | O | O | O | O | … |
| Dental care: pediatric / specialized dentist | O | O | O | O | O | O | … |
| General practitioner | O | O | O | O | O | O | … |
| Palliative care | O | O | O | O | O | O | … |

10b. Which additional elements of healthcare or disciplines should be reflected in the set of indicators? (in other words, do you miss elements of healthcare / disciplines in the above list and how important do you consider them to be?

PLEASE NOTE: this question (still) concerns adults with Down syndrome.

(If you do not want to add elements of healthcare / disciplines, please click “next”).

| Add healthcare element(s) below | Very important | Important | Neutral | Not that important | Not important at all | Explain your answer (optional) |
| --- | --- | --- | --- | --- | --- | --- |
| … | O | O | O | O | O | … |
| … | O | O | O | O | O | … |
| … | O | O | O | O | O | … |
| … | O | O | O | O | O | … |
| … | O | O | O | O | O | … |

11. Healthcare for adults (18 years and older) with Down syndrome:

11a. Do you think the set of indicators should be able to measure adherence to a multidisciplinary guideline for healthcare for adults with Down syndrome?

NOTE: A multidisciplinary guideline for healthcare for adults with Down syndrome is currently being developed (Similar to the guideline for children). This question concerns the future use of this guideline.

|  | Very important | Important | Neutral | Not that important | Not important at all | I don’t know |
| --- | --- | --- | --- | --- | --- | --- |
| The indicator set should be able to measure adherence to a multidisciplinary guideline describing healthcare for adults with Down syndrome. | O | O | O | O | O | O |

[IF answer to 11a. was “I don’t know”, “not important at all”, or “not that important”:]

11b. Please explain your answer (optional)

…

[IF answer to 11a. was “very important”, “important”, or “neutral”:]

11c. A guideline describing multidisciplinary healthcare for adults with Down syndrome is currently being developed. Which healthcare elements should this guideline contain AND should be reflected in the indicator set? (more than one answer possible)

- Visit to an ID physician (frequency with which an adult with down syndrome (and his/her relatives) visits an ID physician)
- Visit to a Down team (frequency with which an adult with down syndrome (and his/her relatives) visits a Down team)
- Visit to an ENT-physician (frequency with which an adult with down syndrome (and his/her relatives) visits an ENT-physician)
- Visit to an ophthalmologist (frequency with which an adult with down syndrome (and his/her relatives) visits an ophthalmologist)
- Visit to an orthoptist (frequency with which an adult with down syndrome (and his/her relatives) visits an orthoptist)
- Visit to a dentist (frequency with which an adult with down syndrome (and his/her relatives) visits a dentist)
- Visit to an orthodontist (frequency with which an adult with down syndrome (and his/her relatives) visits an orthodontist)
- Visit to a physiotherapist (frequency with which an adult with down syndrome (and his/her relatives) visits a physiotherapist)
- Visit to a speech therapist (frequency with which an adult with down syndrome (and his/her relatives) visits a speech therapist)
- Visit to a psychologist (frequency with which an adult with down syndrome (and his/her relatives) visits a psychologist)
- Thyroid screening
- Celiac disease screening
- Palliative care
- Other elements / topics, namely: ……
- I don’t know

[IF answer to 11a. was “very important”, “important”, or “neutral”:]

11d. Please explain your answers concerning a future guideline for healthcare for adults with Down syndrome. (optional)

…

12. Healthcare for adults (18 years and older) with Down syndrome:

12a. Do you think that the set of indicators should include indicators reflecting the use of additional guidelines, protocols and / or quality standards?

For example: screening lists (like “Health Watch”), guidelines in your field of expertise, or from the Dutch quality framework for care for people with intellectual disabilities, or other)

|  | Very important | Important | Neutral | Not that important | Not important at all | I don’t know |
| --- | --- | --- | --- | --- | --- | --- |
| The indicator set should be able to measure adherence to additional guidelines / protocols / quality standards | O | O | O | O | O | O |

[IF answer to 12a. was “very important”, “important”, or “neutral”:]

12b. Which additional guidelines / protocols / quality standards?

(For example: guidelines in your field of expertise, or from the Dutch quality framework for care for people with intellectual disabilities, or other)

…

12c. Please explain (optional)

…

13. Healthcare for adults (18 years and older) with Down syndrome:

13a. Which quality domains or topics should be reflected in the set of indicators?

|  | Very important | Important | Neutral | Not that important | Not important at all | Explain your answer (optional) |
| --- | --- | --- | --- | --- | --- | --- |
| Clinical outcomes  (for example: improved serology, cardiac function, BMI) | O | O | O | O | O | … |
| Outcomes relevant for the patient (e.g. quality of life, daily functioning, participation) | O | O | O | O | O | … |
| Coordination *within* an organization or department (for example: presence of a coordination, multidisciplinary consultation) | O | O | O | O | O | … |
| Coordination *between* healthcare professionals of different organizations and sectors (for example: between healthcare professionals in primary care, secondary care, or social care) | O | O | O | O | O | … |
| Transition 18- to 18+  (transition / transfer from pediatric to adult healthcare) | O | O | O | O | O | … |
| Findability (information on available healthcare providers and their differences) | O | O | O | O | O | … |
| Accessibility (for example: ease of making appointments, waiting time, geographical location) | O | O | O | O | O | … |
| Expertise (for example knowledge present among healthcare professionals) | O | O | O | O | O | … |
| Person-centeredness (for example: patient-professional relation, communication, taking into account life phase and preferences of patient and parents) | O | O | O | O | O | … |
| Equality | O | O | O | O | O | … |

13b. Which additional quality domains or topics should be reflected in the set of indicators? (in other words, do you miss quality domains or topics in the above list and how important do you consider them to be?

PLEASE NOTE: this question (still) concerns adults with Down syndrome.

(If you do not want to add quality domains or topics, please click “next”).

| Add quality domains or topics below | Very important | Important | Neutral | Not that important | Not important at all | Explain your answer (optional) |
| --- | --- | --- | --- | --- | --- | --- |
| … | O | O | O | O | O | … |
| … | O | O | O | O | O | … |
| … | O | O | O | O | O | … |
| … | O | O | O | O | O | … |
| … | O | O | O | O | O | … |

The following questions concern healthcare for both children and adults with Down syndrome.

14. Indicators for healthcare for people with Down syndrome in practice.

14a. What is the most important purpose of a set of indicators?

| Indicators have to provide information in order to… | Very important | Important | Neutral | Not that important | Not important at all | Explain your answer (optional) |
| --- | --- | --- | --- | --- | --- | --- |
| …map available healthcare, providing people with Down syndrome and their family with information on where to find good healthcare. | O | O | O | O | O | … |
| …minimalize geographical differences in healthcare supply and quality, leading to an even distribution of quality across healthcare providers in the Netherlands. | O | O | O | O | O | … |
| …improve healthcare for people with Down syndrome in the Netherlands. | O | O | O | O | O | … |
| …improve healthcare for people with Down syndrome provided in my hospital / department / practice / organization. | O | O | O | O | O | … |
| …inform development of guideline(s) for healthcare for people with Down syndrome. | O | O | O | O | O | … |
| …provide input for healthcare purchasing by health insurers | O | O | O | O | O | … |
| …inspect and control quality and safety of healthcare for people with Down syndrome (by the national inspectorate) | O | O | O | O | O | … |
| …inform (national) policy concerning healthcare for people with Down syndrome | O | O | O | O | O | … |
| …enable scientific research. | O | O | O | O | O | … |

14b. Please indicate whether you think the set of indicators should serve additional purposes and indicate the importance of these purposes. (in other words, do you miss purposes in the above list?)

(If you do not want to add purposes, please click “next”).

| Add purposes below | Very important | Important | Neutral | Not that important | Not important at all | Explain your answer (optional) |
| --- | --- | --- | --- | --- | --- | --- |
| … | O | O | O | O | O | … |
| … | O | O | O | O | O | … |
| … | O | O | O | O | O | … |
| … | O | O | O | O | O | … |
| … | O | O | O | O | O | … |

The following questions (still) concern healthcare for both children and adults with Down syndrome.

15. Indicators for healthcare for people with Down syndrome in practice.

15a. Which guideline(s) are you using in your work with people with Down syndrome?

…

15b. Which indicators are you using in your work with people with Down syndrome? Or in what way is quality of healthcare being monitored by your organization?

…

The following questions (still) concern healthcare for both children and adults with Down syndrome.

16. Information source(s) for indicators.

Information from different sources may be used to provide insight into quality of healthcare, such as: information obtained from electronic medical records and from questionnaires for patients. Perhaps, you also register information in an electronic medical record, or your organization asks patients to fill out a questionnaire on experienced care.

16a. On which healthcare elements / disciplines does your organization / practice / department collect quality information?

(more answers possible)

- Down team for children
- Down team for adults
- Physiotherapy
- Speech therapy
- Dietetics
- Occupation therapy
- Podiatry
- Youth care (municipal health service: screening and vaccinations)
- Dental hygiene
- Regular dentistry (primary care)
- Pediatric / specialized dentist
- General practice
- Other healthcare (providers), namely: ….
- My organization / practice / department does not collect any quality information on healthcare for people with Down syndrome.
- Not applicable, I am (currently) not employed by a healthcare organization providing healthcare to people with Down syndrome.

16b. Please explain your answer (optional)

…

16c. On which quality domains / topics does your organization / practice / department collect quality information?

(more answers possible)

- On topics mentioned in the guideline(s) I use
- Transition from pediatric to adult healthcare
- Clinical outcomes (for example: improved serology, cardiac function, BMI).
- Outcomes relevant for the patient (e.g. quality of life, daily functioning, participation)
- Coordination *within* an organization or department (for example: presence of a coordination, multidisciplinary consultation)
- Coordination *between* healthcare professionals of different organizations and sectors (for example: between healthcare professionals in primary care, secondary care, or social care)
- Findability (information on available healthcare providers and their differences)
- Accessibility (for example: ease of making appointments, waiting time, geographical location)
- Expertise of healthcare professionals
- Person-centeredness (for example: patient-professional relation, communication, taking into account life phase and preferences of patient and parents)
- Equality
- Other quality domains / topics, namely: …
- My organization / practice / department does not collect any quality information on healthcare for people with Down syndrome.
- Not applicable, I am (currently) not employed by a healthcare organization providing healthcare to people with Down syndrome.

16b. Please explain your answer (optional)

…

17. Information source(s) for indicators.

For this set of indicators we aim to use information that is already being registered or available as much as possible. However, a possible outcome of this study may be that additional information (not yet being registered / available) is needed for the set of indicators.

17a. Do you think your colleagues (having the same profession) will be willing to register additional information (next to what is already being registered)?

- No, I don’t think so.
- Only if this entails just clicking a few extra boxes in the registration system.
- Yes, I think they will.

17b. Please explain your answer (for example: concerning your ideas on how to collect information)

(optional)

…

18. The final set of indicators

The development of a set of indicators that is applicable to all Down teams in the Netherlands within this research project is a feasible exercise, because Down teams form a delineated part of healthcare for people with Down syndrome. Preferably, we would like to develop indicators for all healthcare providers involved in healthcare for people with Down syndrome. However, we will face several issues, such as:

- Should the set of indicators be applicable to both healthcare professionals with frequent and with occasional contact with people with Down syndrome?
  - If the set would only be applicable to healthcare professionals with frequent contact with people with Down syndrome, how to define and identify these professionals?
  - If the set would also be applicable to healthcare professionals with only occasional contact with people with Down syndrome, is it fair and valid to measure quality of provided care by these professionals? (for example: is it fair to expect each general practitioner to have expertise on Down syndrome?)
- On which level should the set of indicators provide information? (for example: on the level of healthcare professionals, organizations, departments, or on regional or municipal level)
- Where is the needed information to be found? (for example: if the set would be applicable to a region, how to make sure that healthcare providers within that region provide the needed data and who will be responsible to collect and manage these data?)

Please reflect on the above issues. What would you advise us to do?

…

19. Are there additional topics you would like to share with us concerning the set of indicators for healthcare for people with Down syndrome?

…

20. Are there other issues concerning this study (this, and the following questionnaires) you would like to share with us?

…

This is the end of this questionnaire.

By clicking “next” you will complete the questionnaire and you will not be able to adapt your answers any more.

(if you would like to change your answers, please click “back” to go to the answer(s) you would like to change. You will not lose any given answers)

Round 2

Dear participant,

Thank you very much for your willingness to take part in this study. This study concerns quality indicators for measuring quality of healthcare for people with Down syndrome. The aim of this study is to identify potential quality indicators and to reveal how these indicators may be used in practice.

The study involves four rounds: the first one, which is completed, and round two, three and four, which will take place today. Today, you will receive three questionnaires: at 10.00am (the current one), around 01.00pm, and around 15.30pm.

Please complete the current questionnaire (round 2) by 11.00am.

You gave informed consent for participation in this study. Please note that participation is on a voluntary basis. You are free to withdraw from the study at any moment, without an explicit reason.

Please do not hesitate to contact us if you experience any problems [phone number and e-mail address of first author].

Good luck!

Kind regards,

[names of the authors]

Please click “next” to start the questionnaire.

1. Not all participants were able to fill out the first questionnaire (round 1).

Did you complete the first questionnaire?

- Yes
- No

[IF answer to question 1 was “No”]

2. What is your age? (scroll down menu 20-90)

3. What is your gender?

- Male
- Female

4. What is your current professional position?

*If you are currently not employed, please mention this in your answer and indicate the position you have had for the longest period.*

…

5. (approximately) how long did you work / have you been working in this position?

*If you are currently not employed, please indicate how long you worked in the position indicated in the former question.*

…

6a. Professionally, how often are you in contact with people with Down syndrome (children and/or adults)?

*If you are currently not employed, please indicate how often you were professionally in contact with people with Down syndrome in the position mentioned in the former question.*

- (almost) daily
- Weekly
- Monthly
- Biannually
- Annually
- Less than once a year

6b. Explanation related to your profession and/or your contact with people with Down syndrome (questions 3-5)

(optional)

…

Indicators

Based on literature, existing guidelines, previous input of healthcare professionals and interviews with people with Down syndrome, their parents, and support staff, we identified relevant elements of quality of healthcare for people with Down syndrome. The number of elements appeared to be large.

*"An indicator is a ‘measurable element of practice performance (…) that can be used to assess the quality, and hence change in quality, of care provided” (Lawrence et al., 1997). An indicator is a signaling agent: it is not a direct measure of quality, but indicates a certain aspect of healthcare provision, which may be reason for further investigation. (Handleiding indicatorenontwikkeling, Kennisinstituut Medisch Specialisten, 2013)*

Preferably, a small number of indicators provides as much information as possible. In other words, we strive to obtain a good impression of quality of healthcare for people with Down syndrome using only a few indicators. An important reason for this is to limit administrative burden. Therefore, this study aims to select indicators that best reflect healthcare quality, and at the same time, lead to the least administrative burden.

This study

With the current and the following questionnaires, we aim to reveal the topics that are, according to you, relevant for a set of indicators for healthcare for people with Down syndrome. We would also like to get insight into your ideas about how such a set should or could be used in practice. In the questionnaires, the following themes will be addressed:

- Aim and execution of this study
- Quality of healthcare for people with Down syndrome
- Purposes and use of a set of indicators
- Quality domains the set should focus on
- Healthcare disciplines the set should be covering.

Aim and execution of this study

7a. Please indicate the extent to which you agree with the following proposition:

|  | Totally agree | Agree | Neutral | Disagree | Totally disagree |
| --- | --- | --- | --- | --- | --- |
| The questionnaires (this study) have to be used to identify the relevant topics of the set of indicators. The actual development of the indicators (“How to measure quality on these topics?”) has to be done by the researchers and relevant stakeholders. | O | O | O | O | O |

7b. According to you, who should be involved in the development of the set of indicators?

…

7c. Would you be willing to be involved in the further development of the set of indicators?

- Yes
- No
- Maybe (depending on the time investment needed, the planning of the development, etc.)

8. Normally, before a set of indicators is being finalized, a concept version of such a set is presented to experts for consultation.

Which experts do you think should be consulted?

…

9. Finally, the final set of indicators will be presented to the Dutch Healthcare Institute, in order to register the set in a national database of quality instruments for the Dutch healthcare. The institute demands that quality instruments are approved by representative of at least patients / clients, healthcare providers, and health insurers.

9a. Representatives of which patient / client groups or organizations do you think should be involved?

…

9b. Representatives of which healthcare providers do you think should be involved?

…

9c. Representatives of which health insurers do you think should be involved?

…

9d. Representatives of which other groups should be involved? (optional)

…

10a. According to you, what should be the maximum number of indicators in the set? (scroll down menu 1-75)

10b. Please explain your answer. (optional)

…

Quality of healthcare for people with Down syndrome

11. The following propositions concern quality of healthcare for people with Down syndrome.

11a. Please indicate the extent to which you agree with the following propositions:

|  | Totally agree | Agree | Neutral | Disagree | Totally disagree |
| --- | --- | --- | --- | --- | --- |
| 1. An up-to-date multidisciplinary guideline is crucial for quality in healthcare for people with Down syndrome. | O | O | O | O | O |
| 1. Guidelines should always contain quality indicators measuring quality of healthcare. | O | O | O | O | O |
| 1. In theory, guidelines are nice, but in practice, they are barely applicable. | O | O | O | O | O |
| 1. Indicators for healthcare for people with Down syndrome should be based on an up-to-date multidisciplinary guideline. | O | O | O | O | O |
| 1. Indicators do not contribute to quality of healthcare for people with Down syndrome. | O | O | O | O | O |
| 1. Healthcare for children with Down syndrome should strive to enable parents / relatives to decide about healthcare for their child / relative with Down syndrome. | O | O | O | O | O |
| 1. Healthcare for adults with Down syndrome should enable them / their relatives to decide about healthcare they receive. | O | O | O | O | O |
| 1. The purpose of healthcare for people with Down syndrome is to improve quality of life of people with Down syndrome. | O | O | O | O | O |
| 1. Quality of healthcare for people with Down syndrome is highly dependent on the presence of a healthcare professional who coordinates the large number of disciplines involved. | O | O | O | O | O |
| 1. High quality in healthcare for children with Down syndrome can only be achieved if healthcare professionals collaborate in multidisciplinary teams on a permanent basis, which is the case in existing Down teams in the Netherlands. | O | O | O | O | O |
| 1. High quality in healthcare for adults with Down syndrome can only be achieved if healthcare professionals collaborate in multidisciplinary teams on a permanent basis, which is the case in existing Down teams in the Netherlands. | O | O | O | O | O |
| 1. Down teams do not bring any added value to existing clinics for people with intellectual disabilities, having expertise in several syndromes and contact with several healthcare professionals. | O | O | O | O | O |
| 1. Quality of healthcare for people with Down syndrome is highly dependent on the competences of individual healthcare professionals. | O | O | O | O | O |
| 1. Healthcare professionals not having contact with people with Down syndrome on a regular basis cannot be expected to have much expertise in Down syndrome. | O | O | O | O | O |
| 1. General practitioners and other healthcare professionals having only occasional contact with people with Down syndrome do not need to have much knowledge on Down syndrome, as long as they know where to find information, or to which healthcare professionals they can make referrals. | O | O | O | O | O |

11b. Please explain your answers regarding the above propositions. (optional)

…

Purposes and use of the set of indicators

Participants in the previous round considered the following purposes for the set of indicators (very) important:

- Providing insight into available healthcare (providers)
- Minimalize geographical differences in healthcare supply and quality, leading to an even distribution of quality across healthcare providers in the Netherlands.
- Improve healthcare for people with Down syndrome in the Netherlands.
- Inform development of guideline(s) for healthcare for people with Down syndrome.
- Inspect and control quality and safety of healthcare for people with Down syndrome (by the national inspectorate)

The opinions of the participants concerning the following purposes were diverse:

- Improve healthcare for people with Down syndrome provided in my hospital / department / practice / organization.

Participants considered the following purposes not important:

- Provide input for healthcare purchasing by health insurers
- Inform (national) policy concerning healthcare for people with Down syndrome
- Enable scientific research.

It is remarkable that opinions of participants in the previous round concerning “Improve healthcare for people with Down syndrome provided in my hospital / department / practice / organization” were very divided, given that they indicated the other propositions on healthcare improvement as being important.

12. In order to find out more about your opinion and its details, and to provide you with the opportunity to revise your previous answers, the following propositions are presented to you.

12a. Please indicate the extent to which you agree with the following propositions:

|  | Totally agree | Agree | Neutral | Disagree | Totally disagree |
| --- | --- | --- | --- | --- | --- |
| 1. The set of indicators has to provide information in order to inform development of guideline(s) for healthcare for people with Down syndrome. | O | O | O | O | O |
| 1. The set of indicators has to provide information in order to improve healthcare for people with Down syndrome provided in my hospital / department / practice / organization. | O | O | O | O | O |
| 1. The set of indicators has to provide insight into which hospital / department / practice / organization provides good healthcare and which does not. | O | O | O | O | O |

In order to obtain more insight into your opinion regarding “Providing insight into available healthcare (providers)“ we prepared the following propositions.

12b. Please indicate the extent to which you agree with the following propositions:

|  | Totally agree | Agree | Neutral | Disagree | Totally disagree |
| --- | --- | --- | --- | --- | --- |
| 1. The set of indicators has to provide people with DS, their parents, and support staff with information on where to find suitable healthcare. | O | O | O | O | O |
| 1. The set of indicators has to provide healthcare professionals with information on where to find good healthcare for people with Down syndrome (for making referrals). | O | O | O | O | O |
| 1. The set of indicators has to provide people with DS, their parents, and support staff with information on their rights in healthcare. |  |  |  |  |  |
| 1. The set of indicators has to provide people with DS, their parents, and support staff with information on available NON-medical care (such as family support). | O | O | O | O | O |
| 1. The set of indicators has to provide healthcare professionals with information on available NON-medical care (such as family support). | O | O | O | O | O |

12c. We would also like to present the following propositions to you (again).

| The set of indicators should provide information suitable as input or guidance for … | Very important | Important | Neutral | Not that important | Not important at all |
| --- | --- | --- | --- | --- | --- |
| 1. … healthcare purchasing by health insurers | O | O | O | O | O |
| 1. … inspection and control of quality and safety (by the national inspectorate) | O | O | O | O | O |
| 1. … (national) policy | O | O | O | O | O |
| 1. … scientific research | O | O | O | O | O |

Do you miss purposes? Would you like to explain your answers?

12d. Please indicate whether you have missed purposes in the above list. You may also want to explain your answers (concerning purposes of the set of indicators), please do so below. (optional)

I would like to add the following purposes:

…

I would like to explain my answers:

…

Quality domains

In the previous round, participants indicated which quality domains or topics they thought the set of indicators should cover, and which domains or topics the considered less important.

Participants in the previous round considered the following quality domains important for the set of indicators:

| Quality domain | Participants’ comments |
| --- | --- |
| Clinical outcomes  (for example: improved serology, heart function, BMI) | *Important to monitor.*  *Does not necessarily indicate quality.* |
| Outcomes relevant for the patient (e.g. quality of life, daily functioning, participation) | *Is very important for a happy feeling.*  *Does not necessarily indicate quality of provided care, is also influenced by other factors.* |
| Coordination *within* an organization or department | *Collaboration is very important.* |
| Coordination *between* healthcare professionals of different organizations and sectors | *Important for making the right, and timely, referrals.* |
| Transition 18- to 18+ | *Currently, this does not run smoothly, there is a ‘gap’ after 18.*  *It is important that information does not need to be gathered anew.* |
| Expertise | *Healthcare professionals need to know where expertise is to be found.* |
| Person-centeredness | *But consider privacy.* |

Participants considered the following quality domains less important:

| Quality domain | Participants’ comments |
| --- | --- |
| Findability | *It is important for getting the right care.*  *May be the purpose of a potential set of indicators.* |
| Accessibility | *Important, but difficult to improve.* |
| Equality | *Equality is taken for granted* |

The following quality domains were added:

| Quality domain | Participants’ comments |
| --- | --- |
| Empowerment, self-management | *Also consider explicit involvement of legal representative of the person with Down syndrome.* |
| Participation in society | *Quality of housing* |
| Religion, spirituality | *Leisure, work, school, daily activities* |

1. The above topics / domains are presented to you again, with a few adaptations (based on the comments given by the participants).

13a. Please indicate how important you consider the following quality domains for the set of indicators.

| The set of indicators should provide insight into… | Very important | Important | Neutral | Not that important | Not important at all |
| --- | --- | --- | --- | --- | --- |
| … how people with DS in the Netherlands are doing (for instance by providing information on prevalence of disease, overweight, quality of life, functioning or participation in society) | O | O | O | O | O |
| … adherence to guidelines (for example by monitoring whether screenings mentioned in guidelines are carried out in time). | O | O | O | O | O |
| … coordination and collaboration within AND between organizations. | O | O | O | O | O |
| … transition from pediatric healthcare (until the age of 18) to adult healthcare (starting at the age of 18). | O | O | O | O | O |
| … effectiveness (such as effect of interventions, expertise of healthcare professionals, timely recognition of health problems). | O | O | O | O | O |
| … person-centeredness (such as interaction between healthcare professionals and person with Down syndrome, healthcare tailored to the desires and possibilities of people with Down syndrome). | O | O | O | O | O |
| … safety of provided healthcare for people with Down syndrome. | O | O | O | O | O |

Do you miss quality domains or topics? Would you like to explain your answers?

13b. Please indicate whether you have missed quality domains or topics in the above list. You may also want to explain your answers (concerning quality domains or topics the set of indicators should cover), please do so below. (optional)

Please note: this question only concerns quality domains / topics. Healthcare elements and disciplines to be covered by the set of indicators will be addressed further on in the questionnaire.

I would like to add the following quality domains / topics:

…

I would like to explain my answers:

…

Structure, process and outcome indicators

Quality of healthcare is often described in terms of healthcare structure, care processes, and health outcomes. Accordingly, indicators can be grouped into structure, process and outcome indicators. By *structure* of healthcare we mean the healthcare system: availability of facilities and qualified staff, rules and regulations, protocols, and financial means (including health insurance). Care *processes* are: all actions taking place between patients and healthcare professionals, both technical interventions (such as measuring blood pressure), and interactions between professional and patient (such as communication). An often-used measure for quality of healthcare is adherence to guidelines. *Outcomes* of healthcare reflect the result of provided healthcare: whether the patient’s situation has improved or not. Examples of outcome measures are presence/absence of disease, increase/decrease of complaints, quality of life. An outcome indicator is a measure for the total care path, including the processes and structures, which contributed to the outcome.

What type of indicators do you think the set should include? (structure, process, outcome indicators?)

Below you can indicate the ideal proportion of structure, process, and outcome indicators in the set. You can indicate this by dividing 60 points over the three types of indicators.

For example: If you think the set of indicators should merely consist of process indicators, you should allocate 60 points to ‘Process indicators’. If you think that the number of structure, process and outcome indicators should be equal, you should allocate 20 point to each type of indicator.

1. You may divide the points however you like, as long as the sum of the points is 60.

Structure (scroll down: 0-60)

Process (scroll down: 0-60)

Outcome (scroll down: 0-60)

Total (automated sum of scores)

Healthcare elements or disciplines the set of indicators should cover

In the previous round, we asked participants which healthcare elements or disciplines they considered important to be covered by the set of indicators. An overview of elements or disciplines that were considered important and the ones that were considered less important is presented below. We also provide insight into healthcare elements or disciplines that were added by the participants in the previous round. The left column concerns healthcare for children with Down syndrome, the right column concerns healthcare for adults with Down syndrome. We also provide a summary of given comments.

The following healthcare elements / disciplines were considered important according to the participants in the previous round:

| Healthcare elements / disciplines for CHILDREN with Down syndrome | Healthcare elements / disciplines for ADULTS with Down syndrome |
| --- | --- |
| Down team  *(“should be available for all children with Down syndrome”; “healthcare professionals need to collaborate”; “contributes to efficiency”)* | Down team  *(“Much room for improvements”)* |
| Pediatrician  *(“essential as part of a Down team, but also as stand-alone discipline”)* | ID physician  *(“the number of ID physicians is too low”)* |
| Physiotherapy  *(“high prevalence of movement problems”; “plays an important advisory role”)* |  |
| Speech therapy  *(“has a positive influence on other health problems”)* |  |
| Dietetics  *(“Important to acquire a good eating pattern and to prevent overweight”)* | Dietetics |
| Psychological care  *(“should be available to everyone”; “there is too less uniformity in psychological care”)* | Psychological care |
| General practitioner  *(“primary contact in healthcare”; “has little knowledge on Down syndrome”)* | General practitioner  *(“primary contact in healthcare”; “has little knowledge on Down syndrome”)* |
| ENT physician | Palliative care  *(“it is important to address this in time”; “including dementia and functional decline”)* |
| Thyroid screening |  |
| Cardiac ultrasound |  |

The following healthcare elements / disciplines were considered less important according to the participants:

| Healthcare elements / disciplines for CHILDREN with Down syndrome | Healthcare elements / disciplines for ADULTS with Down syndrome |
| --- | --- |
| Occupational therapy  *(“brings an added value as compared to physiotherapy and is deployed too little”)* | Occupational therapy  *(“important for general daily activities”)* |
| Podiatrist  *(“high prevalence of feet and walking problems”; “someone should coordinate aids and treatment”)* | Podiatrist  *(“high prevalence of feet and walking problems”; “someone should coordinate aids and treatment”; “pain, inactivity, overweight, and complaints are often caused by problems related to feet/walking/shoes”)* |
| Dermatology  *(“skin problems generally start from puberty, and often receive insufficient attention”)* | Dermatology  *(“more important than for children”)* |
| Youth care  *(“important for vaccinations, coordination and integration at school”)* | Physiotherapy  *(“only when needed”)* |
| Dental care  *(“dental hygiene, regular dental care and specialised dental care are important, but regular specialised care is desirable”; “had impact on other health problems”)* | Dental care  *(“dental hygiene, regular dental care and specialised dental care are important, but regular specialised care is desirable”; “had impact on other health problems”)* |
| Celiac disease screening | Celiac disease screening |
|  | Thyroid screening |
|  | Speech therapy  *(“Improvements are always possible”; “communication, speech, and language are important prerequisites for functioning”)* |
|  | ENT-physician |
|  | Ophthalmology / orthoptist |

The following healthcare elements / disciplines were added by the participants:

| Healthcare elements / disciplines for CHILDREN with Down syndrome | Healthcare elements / disciplines for ADULTS with Down syndrome |
| --- | --- |
| (pediatric) cardiology |  |
| ID physician  *(“in case of complex behaviors or sleeping problems”)* |  |
| Audiology / hearing screening  *(“an ENT-physician is not always needed”)* | Audiology / hearing screening  *(“an ENT-physician is not always needed”)* |
| Child psychiatry | Psychiatry  *(“related to depression/anxiety”; “where to find suitable care?”)* |
| Orthopedics  *(“screening of knees and hips”)* | Orthopedics  *(“screening of feet, knees, and hips”)* |
| Pediatric rehabilitation specialist |  |
| Physical activity professional  *(“for developing a healthy exercise pattern”)* |  |
| Multidisciplinary sleeping research team  *(“related to sleeping apnea”)* | Multidisciplinary sleeping research team  *(“related to sleeping apnea”)* |
| Ophthalmology / orthoptist  *(“important for daily functioning and vision development”)* | Optometrist  *(“complementary to ophthalmology / orthoptist”)* |
| Centre of expertise for blind people |  |
| Specialized (ID) nurse |  |
| Organizations for people with ID |  |
| Support staff  *(“for children not living with their parents”)* |  |
| Diabetes screening | Diabetes screening |
| Sexuality / puberty / contraception |  |
| Family support  *(“professional guidance concerning ‘Early intervention’ / coping with a child with Down syndrome / choosing schools / respite care / local community”)* | Case manager / mentor  *(“has to coordinate care, because parents of adults with Down syndrome are not able to do that anymore”)* |

1. Which healthcare disciplines / elements should be covered by the set of indicators?

On the previous page, we presented healthcare disciplines / elements that, according to the participants in the previous round, should be covered by the set of indicators. Here we present these disciplines / elements again, including newly added disciplines / elements. Please keep in mind that the set of indicators should be as compact and concise as possible. We ask you to indicate only those elements / disciplines as “(very) important” if you think these are crucial for obtaining insight into healthcare for people with Down syndrome.

15a. Which elements of healthcare should be reflected in the set of indicators?

Please indicate this for children (left column) and for adults (right column).

|  | CHILDREN | | | | | ADULTS | | | | |
| --- | --- | --- | --- | --- | --- | --- | --- | --- | --- | --- |
|  | Very important | Important | Neutral | Not that important | Not important at all | Very important | Important | Neutral | Not that important | Not important at all |
| Down team  (a coordinated team of collaborating multidisciplinary healthcare providers collaborating for children or adults with down syndrome) | O | O | O | O | O | O | O | O | O | O |
| Pediatrician | O | O | O | O | O | O | O | O | O | O |
| ID physician | O | O | O | O | O | O | O | O | O | O |
| Physiotherapy | O | O | O | O | O | O | O | O | O | O |
| Speech therapist | O | O | O | O | O | O | O | O | O | O |
| Dietician | O | O | O | O | O | O | O | O | O | O |
| Occupational therapy | O | O | O | O | O | O | O | O | O | O |
| Podiatrist | O | O | O | O | O | O | O | O | O | O |
| Dermatology | O | O | O | O | O | O | O | O | O | O |
| Mental healthcare | O | O | O | O | O | O | O | O | O | O |
| Youth healthcare (municipal health service) | O | O | O | O | O | O | O | O | O | O |
| Dental care: Dental hygienist, regular dentist (primary care), specialized dentist, and orthodontist | O | O | O | O | O | O | O | O | O | O |
| General practitioner | O | O | O | O | O | O | O | O | O | O |
| Care for dementia and functional decline | O | O | O | O | O | O | O | O | O | O |
| Palliative care | O | O | O | O | O | O | O | O | O | O |
| Cardiology | O | O | O | O | O | O | O | O | O | O |
| Rehabilitation | O | O | O | O | O | O | O | O | O | O |
| Orthopedics | O | O | O | O | O | O | O | O | O | O |
| Physical activity professional | O | O | O | O | O | O | O | O | O | O |
| ENT-specialist | O | O | O | O | O | O | O | O | O | O |
| Audiology / hearing screening | O | O | O | O | O | O | O | O | O | O |
| Ophthalmology / orthoptist | O | O | O | O | O | O | O | O | O | O |
| Optometrist | O | O | O | O | O | O | O | O | O | O |
| Centre of expertise for blind people | O | O | O | O | O | O | O | O | O | O |
| Multidisciplinary sleeping research team | O | O | O | O | O | O | O | O | O | O |
| Screening for celiac disease | O | O | O | O | O | O | O | O | O | O |
| Screening for thyroid disease | O | O | O | O | O | O | O | O | O | O |
| Screening for diabetes | O | O | O | O | O | O | O | O | O | O |
| ID specialized nurse / practice nurse | O | O | O | O | O | O | O | O | O | O |
| Support staff in living facilities | O | O | O | O | O | O | O | O | O | O |
| Professional family support | O | O | O | O | O | O | O | O | O | O |
| Case manager / mentor | O | O | O | O | O | O | O | O | O | O |

Do you miss healthcare disciplines or elements? Would you like to explain your answers?

15b. Please indicate whether you have missed healthcare elements or disciplines in the above list. You may also want to explain your answers (concerning healthcare elements or disciplines the set of indicators should cover), please do so below. (optional)

I would like to add the following healthcare elements or disciplines:

…

I would like to explain my answers:

…

1. The following propositions also concern the healthcare elements or disciplines that should be covered by the set of indicators.

16a. Please indicate the extent to which you agree with the following propositions:

(and explain your answers if you like)

|  | Totally agree | Agree | Neutral | Disagree | Totally disagree |
| --- | --- | --- | --- | --- | --- |
| Healthcare elements or disciplines are more important for the set of indicators when more people with Down syndrome need them. | O | O | O | O | O |
| Healthcare elements or disciplines are more important for the set of indicators when they contribute more to quality of life of people with Down syndrome. | O | O | O | O | O |
| Healthcare elements or disciplines are more important for the set of indicators when there are more providing professionals. | O | O | O | O | O |
| Healthcare elements or disciplines are more important for the set of indicators when there are more doubts about the quality of the element / discipline. | O | O | O | O | O |
| Healthcare for people with Down syndrome is multidisciplinary. Therefore, the set of indicators should cover all disciplines involved in healthcare for people with Down syndrome. | O | O | O | O | O |
| Relevance of healthcare elements / disciplines depends on the life phase of a person with Down syndrome. Accordingly, each life phase needs different indicators. | O | O | O | O | O |

16b. Please explain you answers to the above propositions. (optional)

…

This is the end of questionnaire 2.

Be aware: after clicking “next”, you are not able to adapt your answers anymore!

(if you would like to change your answers, please click “back” to go to the answer(s) you would like to change. You will not lose any given answers)

Round 3

Dear participant,

This morning you completed the questionnaire of round 2 of the study. Thanks! In the current questionnaire we present themes similar to the ones in the previous questionnaire(s). In the current questionnaire, the themes on which no consensus was achieved among the participants in the previous round are addressed, and themes on which more detailed information is needed.

Please complete the current questionnaire (round 3) by 2.15 pm.

You will receive the next (and last) questionnaire at 3.30 pm.

You gave informed consent for participation in this study. Please note that participation is on a voluntary basis. You are free to withdraw from the study at any moment, without an explicit reason.

Please do not hesitate to contact us if you experience any problems [phone number and e-mail address of first author].

Good luck!

Kind regards,

[names of the authors]

Please click “next” to start the questionnaire.

Aims and execution of the study

1. A set of indicators should not be developed without involving:

- Clients (people with Down syndrome)
- Parents / relatives
- Support staff or healthcare professionals
- Health insurers

Purposes and use of the set of indicators

Based on the outcomes of the previous rounds, we formulated the following questions:

2. How could the indicators provide information that could be used by people with Down syndrome and their relatives for choosing suitable healthcare, without naming healthcare professionals?

…

3. How could the indicators provide information that could be used by healthcare organizations or professionals to improve provided care, without naming healthcare professionals and organizations?

…

4. From the previous round, it appeared that participants considered “providing information that enables improvements in care provision by my organization” an important purpose of the set of indicators.

How do you think the information provided by the set of indicators should be used?

4a. Please indicate the extent to which you agree with the following propositions and, if desired, please add purposes of the set of indicators.

|  | Totally agree | Agree | Neutral | Disagree | Totally disagree |
| --- | --- | --- | --- | --- | --- |
| 1. Information provided by the indicators should be suitable for short-term evaluations on the level of patients/clients. | O | O | O | O | O |
| 1. Information provided by the indicators should be suitable for interdisciplinary evaluation. | O | O | O | O | O |
| 1. Information provided by the indicators should be suitable for clinical-epidemiological research. | O | O | O | O | O |
| 1. Information provided by the indicators should be suitable as input for adjusting protocols. | O | O | O | O | O |

4b. Which additional purposes, concerning use of indicators for healthcare improvements, would you like to add? Please indicate below: (optional)

…

5. From the previous round, it appeared that participants considered “providing information as input for healthcare purchasing by health insurers” and “providing information as input for inspection and review” important purposes for the set of indicators. More detailed purposes are formulated below.

5a. Please indicate the extent to which you agree with the following propositions and, if desired, please add purposes for the indicators.

|  | Totally agree | Agree | Neutral | Disagree | Totally disagree |
| --- | --- | --- | --- | --- | --- |
| 1. Information provided by the indicators should be suitable as input for negotiations about healthcare purchasing. | O | O | O | O | O |
| 1. Information provided by the indicators should be suitable as input for contracting healthcare providers by health insurers. | O | O | O | O | O |
| 1. Information provided by the indicators should be suitable for assessment of performance of professionals and rewards. | O | O | O | O | O |
| 1. Information provided by the indicators should be suitable as input for inspection by the national inspectorate. | O | O | O | O | O |
| 1. Information provided by the indicators should be suitable as input for review and control by the supervisory board. | O | O | O | O | O |

5b. Which additional purposes, concerning use of indicators by health insurers or for control, would you like to add? Please indicate below: (optional)

…

6. Detail and level of the set of indicators

6a. Please indicate the extent to which you agree with the following propositions.

|  | Totally agree | Agree | Neutral | Disagree | Totally disagree |
| --- | --- | --- | --- | --- | --- |
| 1. The set of indicators should be modular in order to allow users to choose which information they would like to register. | O | O | O | O | O |
| 1. Next to a general set containing indicators of all disciplines, there should be an elaborated set per discipline. | O | O | O | O | O |
| 1. The set should provide quality information on organizational / departmental level. | O | O | O | O | O |
| 1. The set should provide quality information on the level of individual professionals. | O | O | O | O | O |
| 1. There should be a quality mark for professionals / organizations specialized in Down syndrome. | O | O | O | O | O |
| 1. Healthcare organizations / departments should publish quality information on their websites. | O | O | O | O | O |
| 1. The set of indicators should only include healthcare professionals with frequent contact with people with DS. | O | O | O | O | O |
| 1. Joining the set of indicators should be voluntary and could be an opportunity for healthcare providers to display their expertise. | O | O | O | O | O |

6b. Please explain your answers concerning the above propositions. (optional)

…

7. From the previous round, it appeared that participants considered the following quality domains and topics important to be covered by the set of indicators:

- “How people with Down syndrome in the Netherlands are doing”
- “Adherence to guidelines”
- “Coordination and collaboration within and between organizations”
- “Transition from pediatric healthcare to adult healthcare”
- “Effectiveness”
- “Person-centeredness”
- “Safety”.

We now elaborate on these issues and ask you to indicate which (more specific) quality domains and topics you think should be covered by the set of indicators.

Concerning “How people with Down syndrome in the Netherlands are doing”

7a. How important do you think it is that the set provides insight into…

|  | Very important | Important | Neutral | Not that important | Not important at all |
| --- | --- | --- | --- | --- | --- |
| …whether burden for the social environment is taken into account | O | O | O | O | O |
| …whether caregivers have the feeling to have control over the healthcare of the person with Down syndrome. | O | O | O | O | O |
| … autonomy of the person with Down syndrome. | O | O | O | O | O |
| … daily functioning of a person with Down syndrome. | O | O | O | O | O |
| … quality of life of a person with Down syndrome. | O | O | O | O | O |
| … participation in society of a person with Down syndrome (for instance: in relation to work, school, leisure, daily activity centers). | O | O | O | O | O |
| … personal development of a person with Down syndrome, such as motor skills, and sensory, cognitive and speech development. | O | O | O | O | O |
| … experienced health problems, such as pain and fatigue. | O | O | O | O | O |
| … measurable physical health (e.g. BMI, blood tests). | O | O | O | O | O |
| … physical health, as experienced by the person with Down syndrome. | O | O | O | O | O |
| … mental health, as experienced by the person with Down syndrome. | O | O | O | O | O |

7b. Please explain your answers to the above propositions and add topics: (optional)

Please note: healthcare elements / disciplines to be covered by the set of indicators will follow later on in the questionnaire.

….

Concerning “Coordination and collaboration within and between organizations”

7c. How important do you think it is that the set provides insight into…

|  | Very important | Important | Neutral | Not that important | Not important at all |
| --- | --- | --- | --- | --- | --- |
| … mutual collaboration among healthcare professionals. | O | O | O | O | O |
| … collaboration between healthcare professionals and parents / relatives / mentors. | O | O | O | O | O |
| … mutual agreements among healthcare providers about tasks and responsibilities. | O | O | O | O | O |
| … agreements between healthcare professionals and parents / relatives / mentors about tasks and responsibilities. | O | O | O | O | O |
| … coordination within organizations or departments. | O | O | O | O | O |
| … collaboration and knowledge sharing between professionals from different organizations and disciplines. | O | O | O | O | O |

Concerning “Transition from pediatric healthcare to adult healthcare”

7d. How important do you think it is that the set provides insight into…

|  | Very important | Important | Neutral | Not that important | Not important at all |
| --- | --- | --- | --- | --- | --- |
| …the presence of a transition protocol | O | O | O | O | O |
| … the way in which transition takes place. | O | O | O | O | O |

7e. Please explain your answers to the above propositions and add topics: (optional)

Please note: healthcare elements / disciplines to be covered by the set of indicators will follow later on in the questionnaire.

….

Concerning “Person-centeredness”

7f. How important do you think it is that the set provides insight into…

|  | Very important | Important | Neutral | Not that important | Not important at all |
| --- | --- | --- | --- | --- | --- |
| … whether preferences, values, living situation etc. of the person with Down syndrome are taken into account. | O | O | O | O | O |
| … self-management (for example: a person with Down syndrome learns how to inject insulin). | O | O | O | O | O |
| … shared decision making. | O | O | O | O | O |
| … whether several disciplines can be visited on one day | O | O | O | O | O |
| … the presence of one contact person for a person with Down syndrome / caregivers parents / relatives / mentors. | O | O | O | O | O |
| … whether healthcare is nearby. | O | O | O | O | O |

Concerning “Effectiveness”

7g. How important do you think it is that the set provides insight into…

|  | Very important | Important | Neutral | Not that important | Not important at all |
| --- | --- | --- | --- | --- | --- |
| … cost effectiveness. | O | O | O | O | O |
| … expertise of healthcare professionals. | O | O | O | O | O |
| … timely recognition of health problems. | O | O | O | O | O |

7h. Please explain your answers to the above propositions and add topics: (optional)

Please note: healthcare elements / disciplines to be covered by the set of indicators will follow later on in the questionnaire.

….

Structure, process and outcome indicators

Quality of healthcare is often described in terms of healthcare structure, care processes, and health outcomes. Accordingly, indicators can be grouped into structure, process and outcome indicators. By *structure* of healthcare we mean the healthcare system: availability of facilities and qualified staff, rules and regulations, protocols, and financial means (including health insurance). Care *processes* are: all actions taking place between patients and healthcare professionals, both technical interventions (such as measuring blood pressure), and interactions between professional and patient (such as communication). An often-used measure for quality of healthcare is adherence to guidelines. *Outcomes* of healthcare reflect the result of provided healthcare: whether the patient’s situation has improved or not. Examples of outcome measures are presence/absence of disease, increase/decrease of complaints, quality of life. An outcome indicator is a measure of the total care path, including the processes and structures, which contributed to the outcome.

There was much variation in the answers of the participants in the previous rounds to the question below. Therefore, this question is presented to you again. Do you stick to your previous answer, or would you like to adjust it?

The answers in the previous round were:

|  | Range | Mean | Median |
| --- | --- | --- | --- |
| Structure | 5-30 | 16.2 | 15 |
| Process | 10-30 | 19.1 | 20 |
| Outcome | 10-40 | 24.7 | 25 |

What type of indicators do you think the set should include? (structure, process, outcome indicators?)

Below you can indicate the ideal proportion of structure, process, and outcome indicators in the set. You can indicate this by dividing 60 points over the three types of indicators.

For example: If you think the set of indicators should merely consist of process indicators, you should allocate 60 points to ‘Process indicators’. If you think that the number of structure, process and outcome indicators should equal, you should allocate 20 point to each type of indicator.

8a. You may divide the points however you like, if only the sum of the points is 60.

Structure (scroll down: 0-60)

Process (scroll down: 0-60)

Outcome (scroll down: 0-60)

Total (automated sum of scores)

8b. Please explain your answer regarding the type of indicators below (optional).

…

Healthcare elements / disciplines to be covered by the set of indicators

9a. Which healthcare elements / disciplines are required for (almost) all people with Down syndrome (during at least a certain period) in their lives?

Please mention the one(s) that first come in mind.

…

9b. Which healthcare elements / disciplines are largely contributing to quality of life of people with Down syndrome?

Please mention the one(s) that first come in mind.

…

9c. Providers of which healthcare discipline(s) are largely available? (Hence: people with Down syndrome and their parents / relatives may have many options for choosing a provider)

Please mention the one(s) that first come in mind.

…

9d. For which healthcare discipline(s) do doubts exist concerning the quality of care provided by these disciplines?

Please mention the one(s) that first come in mind.

…

This is the end of questionnaire 3.

Be aware: after clicking “next”, you are not able to adapt your answers anymore!

(if you would like to change your answers, please click “back” to go to the answer(s) you would like to change. You will not lose any given answers)

Round 4

Dear participant,

Earlier today, you completed the questionnaire of round 2 and 3 of the study. Thanks! In the current questionnaire we present themes similar to the ones in the previous questionnaire(s). In the current questionnaire, themes are addressed on which no consensus was achieved among the participants in the previous round(s), and themes on which more detailed information is needed.

Please complete the current questionnaire (round 4) by tomorrow (May 31).

You gave informed consent for participation in this study. Please note that participation is on a voluntary basis. You are free to withdraw from the study at any moment, without an explicit reason.

Please do not hesitate to contact us if you experience any problems [phone number and e-mail address of first author].

Good luck!

Kind regards,

[names of the authors]

Please click “next” to start the questionnaire.

1. There was much variation in the answers of the participants in the previous rounds regarding the following proposition: “The set of indicators has to provide healthcare professionals with information on available NON-medical care”. Therefore, we formulated the next proposition.

1a. Please indicate the extent to which you agree with the following proposition:

|  | Totally agree | Agree | Neutral | Disagree | Totally disagree |
| --- | --- | --- | --- | --- | --- |
| A set of indicators for healthcare for people with Down syndrome should not contain indicators on NON-medical care (such as: daily activity centers, school, leisure time). | O | O | O | O | O |

1b. Please explain your answer (optional).

…

2. From the previous round, it appeared that many participants thought that the set of indicators should consist of modules. However, round 2 did not result in consensus among participants regarding the number of indicators in the set. The answers varied from 5 to 40. Therefore, we formulated a few more questions concerning modules and the number of indicators.

2a. Please indicate the extent to which you agree with the following propositions:

|  | Totally agree | Agree | Neutral | Disagree | Totally disagree |
| --- | --- | --- | --- | --- | --- |
| 1. There should be a basic set of indicators, consisting of indicators that are relevant to all people with Down syndrome. | O | O | O | O | O |
| 2. Next to this basic set, additional modules should be present for specific healthcare or patient groups. | O | O | O | O | O |

[IF answer to proposition 1 is “totally agree”, “agree”, or “neutral”:]

2b. What should be the maximum number of indicators in this basic set?

…

[IF answer to proposition 2 is “totally agree”, “agree”, or “neutral”:]

2c. What should be the maximum number of indicators in each of these additional modules?

…

2d. Which additional modules have most priority for becoming part of the set?

…

3. Please indicate the extent to which you agree with the following propositions:

|  | Totally agree | Agree | Neutral | Disagree | Totally disagree |
| --- | --- | --- | --- | --- | --- |
| Quality information should be public on the organizational level, but not on the provider (personal) level. | O | O | O | O | O |
| Healthcare professionals should themselves decide about public availability of quality information. | O | O | O | O | O |
| Privacy of professionals should be protected just as much as privacy of patients. | O | O | O | O | O |
| Quality of the social system of a person with Down Syndrome (including all his/her caregivers) is crucial in healthcare for people with Down syndrome. | O | O | O | O | O |
| Indicators should stimulate improvement of care and should not judge healthcare professionals. | O | O | O | O | O |
| Professionals should be obliged to register the indicators if they want to be seen as ‘specialized in Down syndrome’. | O | O | O | O | O |
| Professionals wanting to be seen as ‘specialized in Down syndrome’ should be obliged to make their quality information publicly available. | O | O | O | O | O |
| Publishing quality information will not result in long waiting lists since most people with DS / parents will not be willing to travel far for better care. | O | O | O | O | O |
| Well defined outcome indicators are able to provide insight into process and structure too. | O | O | O | O | O |

Practical issues related to collecting quality information

4a. Please indicate the extent to which you agree with the following propositions:

|  | Totally agree | Agree | Neutral | Disagree | Totally disagree |
| --- | --- | --- | --- | --- | --- |
| Standardization and interoperability of electronic medical records needs to be established before quality can be measured. | O | O | O | O | O |
| Burden for people with Down syndrome and their caregivers should be as low as possible when measuring quality. | O | O | O | O | O |
| Burden for healthcare professionals should be as low as possible when measuring quality. | O | O | O | O | O |
| People with Down syndrome (and their parents/relatives) and healthcare professionals should both deliver information for the indicators. | O | O | O | O | O |
| Parents/caregivers should themselves be responsible for documenting and keeping track of needed healthcare for the person with Down syndrome. | O | O | O | O | O |
| A dialogue between healthcare professional and person with Down syndrome should be used as an instrument for measuring customer satisfaction. | O | O | O | O | O |
| An instrument measuring patient experiences or satisfaction should be suitable to be filled out by 80% of the population of people with Down syndrome by themselves. | O | O | O | O | O |
| When people with Down syndrome are not able to provide quality information themselves, their legal representative should decide who is eligible to provide this information. | O | O | O | O | O |

4b. Please explain your answers to the above presented propositions. (optional)

…

This is the end of questionnaire 4, which is the last questionnaire of this study.

Thanks again for your willingness to take part in this study!

Be aware: after clicking “next”, you are not able to adapt your answers anymore!

(if you would like to change your answers, please click “back” to go to the answer(s) you would like to change. You will not lose any given answers)
