# Supplementary material for: Capturing the complexity of healthcare for people with Down syndrome in quality indicators - a Delphi study involving healthcare professionals and patient organisations
Source: BMC Health Serv Res. 2020 Jul 27;20:694. doi: 10.1186/s12913-020-05492-z (PMC7385945; doi:10.1186/s12913-020-05492-z)
Supplement: Supplementary file 2 — Additional file 2: (Supplementary Tables 1–5). Supplementary Tables 1–5: Supplementary Table 1. Extent to which consensus was achieved among participants regarding: Purposes of QIs; Supplementary Table 2. Extent to which consensus was achieved among participants regarding: Quality domains; Supplementary Table 3. Extent to which consensus was achieved among participants regarding: Healthcare services/disciplines; Supplementary Table 4. Preferred number and type of QIs and extent to which consensus was achieved among participants regarding related propositions; Supplementary Table 5. Extent to which consensus was achieved among participants regarding: Information sources and transparency of QIs and practical issues regarding development. Tables indicating the extent to which consensus was achieved among participants regarding different aspects of a QI-set for healthcare for people with DS. Results of the study are largely based on these data. [file 12913_2020_5492_MOESM2_ESM.docx]

**Additional file 2**

**Supplementary Table 1.** Extent to which consensus was achieved among participants regarding: Purposes of QIs

|  | **Round 1** | | **Round 2** | | **Round 3** | |
| --- | --- | --- | --- | --- | --- | --- |
|  | *% 1-2^a^* | *median* | *% 1-2 ^a^* | *median* | *% 1-2 ^a^* | *median* |
| **What should be the purpose of QIs?**  *(How important do you consider the following purposes?)*  **1=very important, 2=important, 3=neutral, 4=not that important, 5=not important at all** | | | | | | |
| Providing people with DS with information for choosing the right care | **93,8** | **1** | **78,6** | **2** |  |  |
| Providing healthcare professionals with information for finding suitable healthcare providers/referrals |  |  | **82,1** | **2** |  |  |
| Reducing differences in provided healthcare in the Netherlands | **81,3** | **2** |  |  |  |  |
| Providing insight into differences between providers |  |  | 60,7 | 2 |  |  |
| Improving healthcare quality for people with DS in the Netherlands | **96,9** | **1** |  |  |  |  |
| Improving healthcare quality provided in own organization (round 1); provide information for doing this (round 2)**^c^** | 65,6 | 2 | **100** | **2** |  |  |
| For short term evaluation of healthcare delivery on the patient level |  |  |  |  | 74,1  **76^b^** | 2 |
| For interdisciplinary evaluation |  |  |  |  | **81,5** | **2** |
| As input for guidelines | **87,5** | **2** | 71,4 | 2 |  |  |
| As input to adjust protocol |  |  |  |  | **92,6** | **2** |
| As input for healthcare purchasing by health insurers | 56,3 | 2 | 42,9 | 3 |  |  |
| As input for negotiating about healthcare purchasing |  |  |  |  | 59,3 | 2 |
| As input for contracting healthcare providers (by insurers) |  |  |  |  | 37 | 3 |
| As input for performance rewards for providers |  |  |  |  | 33,3 | 3 |
| For inspection and review | **75** | **2** |  |  |  |  |
| For inspection by the national inspectorate |  |  | **82,1** | **2** | **88,9** | **2** |
| For review and control by the supervisory board |  |  |  |  | **81,5** | **2** |
| As input for policy | 65,6 | 2 | 60,7 | 2 |  |  |
| For scientific research | 65,6 | 2 | **75** | **2** |  |  |
| For clinical-epidemiological research |  |  |  |  | 51,9 | 2 |
| Other, please add: |  |  |  |  |  |  |
| Providing people with DS with information on their rights in healthcare |  |  | 64,3 | 2 |  |  |
| A purpose of QIs should be to provide people with DS / caregivers with insight into available non-medical care |  |  | 67,9 | 2 |  |  |
| A purpose of QIs should be to provide healthcare professionals with insight into available non-medical care. |  |  | 71,4 | 2 |  |  |

Abbreviations: QI=Quality indicator; DS=Down syndrome.

Empty fields indicate that the topic was not presented to the participants in the concerning round.

***^a^*** %1-2 indicates the percentage of participants that had answered “very important”/”totally agree” (1) or “important”/”agree” (2). If the percentage was ≥ 75 and the median was ≤ 2, there was consensus among the participants. Consensus was indicated in **bold**.

**^b^** Percentage of participants that had answered “very important” (1) or “important” (2) if patient representatives are not included in analysis. The difference was only showed if exclusion of patient representatives resulted in a different conclusion regarding consensus.

^c^ Formulation of purpose was different in round 1 and 2.

^d^ % 4-5, indicating the percentage of participants that had answered “disagree” (4) or “totally disagree” (5).

**Supplementary Table 2.** Extent to which consensus was achieved among participants regarding: Quality domains

|  | **Round 1** | | | | **Round 2** | | | **Round 3** | | **Round 4** | |
| --- | --- | --- | --- | --- | --- | --- | --- | --- | --- | --- | --- |
| **Which quality domains should be covered by the QIs?**  *(How important do you consider the following quality domains for QIs?)*  **1=very important, 2=important, 3=neutral, 4=not that important, 5=not important at all** | | | | | | | | | | | |
|  | **Children** | | **Adults** | | **(Children and adults together)^b^** | | **(Children and adults together) ^b^** | | | **(Children and adults together) ^b^** | |
|  | *% 1-2* ***^a^*** | *median* | *% 1-2* *^a^* | *median* | *% 1-2 ^a^* | *median* | *% 1-2 ^a^* | | *median* | *% 1-2 ^a^* | *median* |
| Coordination within organisations or departments | **81,8** | **2** | **78,8** | **2** |  |  | 70,4 | | 2 |  |  |
| Coordination between organisations or departments | **97** | **2** | **93,9** | **2** |  |  | **100** | | **1** |  |  |
| Coordination within and between organisations or departments |  |  |  |  | **85,7** | **2** |  | |  |  |  |
| Mutual collaboration among healthcare providers |  |  |  |  |  |  | **88,9** | | **2** |  |  |
| Collaboration between healthcare providers and caregivers of people with DS |  |  |  |  |  |  | **88,9** | | **1** |  |  |
| Mutual agreements among healthcare providers about tasks and responsibilities |  |  |  |  |  |  | **81,5** | | **2** |  |  |
| Mutual agreements between healthcare providers and caregivers about tasks and responsibilities |  |  |  |  |  |  | 74,1 | | 2 |  |  |
| Quality of the social system of a person with DS (including all his/her caregivers) |  |  |  |  |  |  |  | |  | 69,2  **75^c^** | 2 |
| Transition from child to adult care | **100** | **2** | **97** | **1** | **96,4** | **1,5** |  | |  |  |  |
| The presence of a transition protocol |  |  |  |  |  |  | **85,2** | | **2** |  |  |
| The way in which transition takes place |  |  |  |  |  |  | **88,9** | | **2** |  |  |
| Whether healthcare is findable | 69,7 | 2 | 72,7 | 2 |  |  |  | |  |  |  |
| Accessibility | 66,7 | 2 | 66,7 | 2 |  |  |  | |  |  |  |
| Safety |  |  |  |  | **78,6** | **2** |  | |  |  |  |
| Equity | 66,7 | 2 | 63,6 | 2 |  |  |  | |  |  |  |
| Expertise | **97** | **1** | **93,9** | **2** |  |  | **85,2** | | **2** |  |  |
| Effectiveness |  |  |  |  | **92,9** | **1,5** |  | |  |  |  |
| Cost effectiveness |  |  |  |  |  |  | 51,9 | | 2 |  |  |
| Timely recognition of health problems |  |  |  |  |  |  | **100** | | **1** |  |  |
| Person-centeredness | **87,9** | **2** | **87,9** | **1** | **85,7** | **2** |  | |  |  |  |
| Whether preferences, values, living situation etc. of the person with DS are taken into account |  |  |  |  |  |  | 66,7 | | 2 |  |  |
| Self-management |  |  |  |  |  |  | 74,1 | | 2 |  |  |
| Shared decision making |  |  |  |  |  |  | **96,3** | | **2** |  |  |
| Whether several disciplines can be visited on one day |  |  |  |  |  |  | 44,4 | | 3 |  |  |
| The presence of one contact person for a person with DS / caregivers |  |  |  |  |  |  | **88,9** | | **2** |  |  |
| Whether healthcare is nearby |  |  |  |  |  |  | **77,8** | | **2** |  |  |
| Clinical outcomes | **84,8** | **2** | **84,8** | **2** |  |  |  | |  |  |  |
| Outcomes relevant for the patient (e.g. quality of life, daily functioning, participation) | **97** | **1** | **97** | **1** | **82,1** | **2** |  | |  |  |  |
| Whether burden for the social environment is taken into account |  |  |  |  |  |  | 44,4 | | 3 |  |  |
| Whether caregivers have the feeling to have control over the healthcare of the person with DS |  |  |  |  |  |  | 66,7 | | 2 |  |  |
| Autonomy of the person with DS |  |  |  |  |  |  | **85,2** | | **2** |  |  |
| Daily functioning |  |  |  |  |  |  | **81,5** | | **2** |  |  |
| Quality of life |  |  |  |  |  |  | **88,9** | | **1** |  |  |
| Participation in society |  |  |  |  |  |  | **77,8** | | **2** |  |  |
| Development |  |  |  |  |  |  | **92,6** | | **2** |  |  |
| Experienced health problems |  |  |  |  |  |  | **96,3** | | **1** |  |  |
| Measurable physical health (e.g. BMI, blood tests) |  |  |  |  |  |  | **96,3** | | **1** |  |  |
| Experienced physical health |  |  |  |  |  |  | **92,6** | | **1** |  |  |
| Experienced mental health |  |  |  |  |  |  | **88,9** | | **2** |  |  |
| Adherence to the Dutch medical guideline for children with DS | **80,6** | **2** |  |  |  |  |  | |  |  |  |
| Adherence to the future Dutch medical guideline for adults with DS**^d^** |  |  | **86,2** | **2** |  |  |  | |  |  |  |
| Adherence to guidelines in general |  |  |  |  | **89,3** | **2** |  | |  |  |  |

Abbreviations: QI=Quality indicator; ID=Intellectual disability; DS=Down syndrome; BMI=Body mass index.

Empty fields indicate that the topic was not presented to the participants in the concerning round.

*^a^* %1-2 indicates the percentage of participants that had answered “very important” (1) or “important” (2). If the percentage was ≥ 75 and the median was ≤ 2, there was consensus among the participants. Consensus was indicated in **bold**.

**^b^** Healthcare for children and adults with DS was taken together in round 2-4, because, based on round 1, we did not expect participants’ opinions about the quality domains to be different regarding children and adults.

**^c^** Percentage of participants that had answered “very important” (1) or “important” (2) if patient representatives are not included in analysis. The difference was only showed if exclusion of patient representatives resulted in a different conclusion regarding consensus.

**^d^** A medical guideline for adults with DS is at this moment being developed in the Netherlands

**Supplementary Table 3.** Extent to which consensus was achieved among participants regarding: Healthcare services/disciplines

|  | **Round 1** | | | | **Round 2** | | | | **Round 4** | |
| --- | --- | --- | --- | --- | --- | --- | --- | --- | --- | --- |
| **About which healthcare services / disciplines should the QIs provide information?**  *(How important do you consider the following services / disciplines for QIs?)*  **1=very important, 2=important, 3=neutral, 4=not that important, 5=not important at all** | | | | | | | | | | |
|  | **Children** | | **Adults** | | **Children** | | **Adults** | |  | |
|  | **% 1-2** *^a^* | **median** | **% 1-2** *^a^* | **median** | **% 1-2** *^a^* | **median** | **% 1-2** *^a^* | **median** |  | |
| Down team child | **100** | **1** |  |  | **100** | **1** | n/a | n/a |  | |
| Down team adult |  |  | **90,6** | **1** | n/a |  | **85,7** | **1** |  | |
| Paediatrician | **100** | **1** |  |  | **85,7** | **1** |  | n/a |  | |
| ID physician |  |  | **100** | **1** | 42,9 | 3 | **96,4** | **1** |  | |
| Physiotherapist | **96,4** | **1,5** | 63 | 2 | **78,6** | **2** | 50 | 2,5 |  | |
| Speech therapist | **96,7** | **1** | 70,4 | 2 | **89,3** | **2** | 60,7 | 2 |  | |
| Dietician | **82,8** | **2** | **81,5** | **2** | 64,3 | 2 | **78,6** | **2** |  | |
| Occupational therapist | 69,2 | 2 | 50 | 2,5 | 50 | 2,5 | 32,1 | 3 |  | |
| Podiatrist | 54,2 | 2 | 50 | 2,5 | 39,3 | 3 | 46,4 | 3 |  | |
| Dermatologist | 48 | 3 | 56,5 | 2 | 21,4 | 3 | 35,7 | 3 |  | |
| Psychologist | **93,3** | **2** | **96,4** | **2** | **92,9** | **2** | **89,3** | **2** |  | |
| Youth healthcare (municipal health service) | 46,2 | 3 |  |  | 28,6 | 3 | n/a | n/a |  | |
| Dental hygienist | **80,8** | **2** | **80,8** | **2** |  | |  | |  | |
| Regular dentist (primary care) | 64 | 2 | 69,2 | 2 |  | |  | |  | |
| Special dentist (secondary care, specialised in special groups) | **79,2** | **2** | **76** | **2** |  | |  | |  | |
| Dentistry together |  |  |  |  | 71,4 | 2 | 71,4 | 2 |  | |
| General practitioner | 71 | 2 | **79,3** | **2** | 53,6 | 2 | 64,3 | 2 |  | |
| Palliative care |  |  | **76** | **2** | n/a |  | 64,3 | 2 |  | |
| Other, please add: |  |  |  |  |  | |  | |  | |
| Care for dementia or decline |  |  |  |  | n/a |  | **85,7** | **2** |  | |
| Cardiologist |  |  |  |  | 71,4 | 2 | 46,4 | 3 |  | |
| Rehabilitation physician |  |  |  |  | 39,3 | 3 | 17,9 | 3 |  | |
| Orthopaedist |  |  |  |  | 39,3 | 3 | 42,9 | 3 |  | |
| Physical education |  |  |  |  | 25 | 3 | 25 | 3 |  | |
| ENT specialist |  |  |  |  | 60,7 | 2 | 35,7 | 3 |  | |
| Hearing screening |  |  |  |  | **89,3** | **2** | **85,7** | **2** |  | |
| Ophthalmologist / orthoptist |  |  |  |  | 67,9 | 2 | 64,3 | 2 |  | |
| Optometrist |  |  |  |  | 46,4 | 3 | 46,4 | 3 |  | |
| Centre of expertise - vision |  |  |  |  | 32,1 | 3 | 35,7 | 3 |  | |
| Centre of expertise - sleeping |  |  |  |  | 17,9 | 3 | 28,6 | 3 |  | |
| Screening for coeliac disease |  |  |  |  | 60,7 | 2 | 46,4 | 3 |  | |
| Screening for thyroid dysfunction |  |  |  |  | 64,3 | 2 | 60,7 | 2 |  | |
| Screening for diabetes mellitus |  |  |  |  | 39,3 | 3 | 60,7 | 2 |  | |
| ID specialised nursing |  |  |  |  | 25 | 3 | 42,9 | 3 |  | |
| Support staff in assisted living facility |  |  |  |  | 46,4 | 3 | 71,4 | 2 |  | |
| Family support |  |  |  |  | **75**  73,1 ^b^ | **2**  2 | 17,9 | 3 |  | |
| Case manager |  |  |  |  | 64,3 | 2 | **85,7** | **2** |  | |
|  |  |  |  |  |  |  |  |  |  | |
| **To what extent do you agree with the following propositions?**  **1=totally agree, 2=agree, 3=neutral, 4=disagree, 5=totally disagree** | | | | | | | | | | |
|  |  |  |  |  | **Children and adults together^c^** | | | |  | |
|  |  |  |  |  | **% 1-2** *^a^* | | **median** | |  | |
| QIs are different for each life phase |  |  |  |  | **89,3** | | **2** | |  | |
| QIs should also cover non-medical disciplines |  |  |  |  |  | |  | | 65,4 | 2 |
| QIs should include all disciplines involved in healthcare for people with DS |  |  |  |  | **78,6** | | **2** | |  | |
| Healthcare elements are more important for the QI-set when: |  |  |  |  |  | |  | |  | |
| - More people with DS need them |  |  |  |  | **96,4** | | **2** | |  | |
| - They contribute more to QoL |  |  |  |  | **100** | | **1** | |  | |
| - There are more providing professionals |  |  |  |  | 32,1 | | 3 | |  | |
| - There are more doubts about the quality of the element |  |  |  |  | **89,3** | | **2** | |  | |

Abbreviations: QI=Quality indicator; ID=Intellectual disability; ENT=Ear nose throat; DS=Down syndrome; QoL=Quality of life.

Empty fields indicate that the topic was not presented to the participants in the concerning round.

*^a^* %1-2 indicates the percentage of participants that had answered “very important”/”totally agree” (1) or “important”/”agree” (2). If the percentage was ≥ 75 and the median was ≤ 2, there was consensus among the participants. Consensus was indicated in **bold**.

^b^ Percentage of participants that had answered “very important” (1) or “important” (2) if patient representatives are not included in analysis. The difference was only showed if exclusion of patient representatives resulted in a different conclusion regarding consensus.

^c^ For these propositions, healthcare for children and adults with DS was taken together, because we did not expect participants’ opinions about these propositions to be different regarding children and adults.

**Supplementary Table 4.** Preferred number and type of QIs and extent to which consensus was achieved among participants regarding related propositions

|  | **Round 2** | **Round 3** | | **Round 4** | |
| --- | --- | --- | --- | --- | --- |
| **How many QIs should the set contain?**  **Median; mean [range]**  **(without patient representatives)** |  |  | |  | |
| Total number | 10; 6,1 [5-40]  (idem) |  | |  | |
| Number in basic set |  |  | | 10; 7,3 [5-30]  (10; 7,9 [5-30]) | |
| Number per module |  |  | | 10; 7 [3-20]  (10; 7,6 [3-20]) | |
|  |  |  | |  | |
| **What type of QIs should the set contain?**  **Mean [range]**  **(without patient representatives)** |  |  | |  | |
| Structure | 16,2 [5-30]  (16,8) | 15,8 [5-30]  (idem) | |  | |
| Process | 19,5 [10-30]  (19) | 21,3 [5-35]  (idem) | |  | |
| Outcome | 24,4 [10-40]  (24,1) | 22,9 [10-50]  (idem) | |  | |
| Distribution Structure : Process : Outcome | 27%:33%:40%  (28%:32%:40%) | 27%:35%:38%  (idem) | |  | |
|  |  | **% 1-2** *^a^* | **median** |  | |
| **To what extent do you agree with the following proposition?**  **1=totally agree, 2=agree, 3=neutral, 4=disagree, 5=totally disagree** |  |  | | **% 1-2** *^a^* | **median** |
| The QI-set should be modular in order to allow users to choose which information should be registered to serve their purpose |  | **81,5** | **2** |  |  |
| Next to a general set containing indicators of all disciplines, there should be an elaborated set per discipline |  | **81,5** | **2** |  |  |
| There should be a basic set of indicators, consisting of indicators that are relevant for all people with DS |  |  |  | **92,3** | **1,5** |
| Next to this basic set, additional modules should be present for specific healthcare or patient groups |  |  |  | **84,6** | **1,73** |
| Well defined outcome indicators are able to provide insight into process and structure too |  |  | | 53,8 | 2 |

Abbreviations: QI=Quality indicator; DS=Down syndrome.

Empty fields indicate that the topic was not presented to the participants in the concerning round.

*^a^* %1-2 indicates the percentage of participants that had answered “totally agree” (1) or “agree” (2). If the percentage was ≥ 75 and the median was ≤ 2, there was consensus among the participants. Consensus was indicated in **bold**.

**Supplementary Table 5.** Extent to which consensus was achieved among participants regarding: Information sources and transparency of QIs and practical issues regarding development

| **To what extent do you agree with the following propositions?**  **1=totally agree, 2=agree, 3=neutral, 4=disagree, 5=totally disagree** | **Round 3** | | **Round 4** | |
| --- | --- | --- | --- | --- |
|  | **% 1-2** *^a^* | **median** | **% 1-2** *^a^* | **median** |
| Standardisation and interoperability of electronic medical records needs to be established before quality can be measured |  |  | 19,2 | 3 |
| Burden for people with DS and their caregivers should be as low as possible when measuring quality |  |  | **76,9** | **2** |
| Burden for healthcare professionals should be as low as possible when measuring quality |  |  | 69,2 | 2 |
| People with DS (+ caregivers) and healthcare professionals should both deliver information for the QIs. |  |  | **100** | **2** |
| Parents/caregivers should themselves be responsible for documenting and keeping track of needed healthcare for the person with DS |  |  | **76,9** | **2** |
| A dialogue between healthcare professional and person with DS should be used as instrument for measuring customer satisfaction |  |  | 73,1  **75^b^** | 2  **2** |
| An instrument measuring patient experiences or satisfaction should be suitable to be filled out by 80% of the DS-population by themselves |  |  | 46,2 | 3 |
| When people with DS are not able to provide quality information themselves, their legal representative should decide who is eligible to provide this information |  |  | **92,3** | **2** |
| Quality information should be available on organisational/ departmental level | **77,8** | **2** |  |  |
| Quality information should be available on provider level | 44,4 | 3 |  |  |
| There should be a quality mark for providers / organisations specialised in DS | 63 | 2 |  |  |
| Healthcare organisations should publish quality information on their websites | **81,5** | **2** |  |  |
| QIs should only measure quality of healthcare provided by healthcare professionals with regular contact with people with DS | 37 | 3 |  |  |
| Applying for joining the QI-set should be voluntary, and could be an opportunity for healthcare providers to display their expertise | 66,7 | 2 |  |  |
| Quality information should be public on the organisational level, but not on provider (personal) level |  |  | **80,8** | **2** |
| Healthcare professionals should themselves decide about public availability of quality information |  |  | **76,9** | **2** |
| Privacy of professionals should be protected just as much as privacy of patients |  |  | **84,6** | **2** |
| QIs should stimulate improvement of care and not judge healthcare professionals |  |  | **92,3** | **2** |
| Professionals should be obliged to register the QIs if they want to be seen as ‘DS-specialised’ |  |  | **88,5** | **1** |
| Professionals wanting to be seen as ‘DS-specialised’ should make their quality information publicly available |  |  | 69,2 | 2 |
| Publishing quality information will not result in long waiting lists since most people with DS / parents will not be willing to travel far for better care. |  |  | 3,8  (61,5 4-5)^c^ | 4 |

Abbreviations: QI=Quality indicator; DS=Down syndrome.

Empty fields indicate that the topic was not presented to the participants in the concerning round.

*^a^* %1-2 indicates the percentage of participants that had answered “totally agree” (1) or “agree” (2). If the percentage was ≥ 75 and the median was ≤ 2, there was consensus among the participants. Consensus was indicated in **bold**.

^b^ Percentage of participants that had answered “totally agree” (1) or “agree” (2) if patient representatives are not included in analysis. The difference was only showed if exclusion of patient representatives resulted in a different conclusion regarding consensus.

^c^ % 4-5, indicating the percentage of participants that had answered “disagree” (4) or “totally disagree” (5).
